# Non-heuristic automatic techniques for overcoming low signal-to-noise-ratio bias of localization microscopy and multiple signal classification algorithm

**DOI:** 10.1038/s41598-018-23374-7

**Published:** 2018-03-21

**Authors:** Krishna Agarwal, Radek Macháň, Dilip K. Prasad

**Affiliations:** 1 Department of Physics and Technology, UiT-The Arctic University of Norway, 9037 Tromsø, Norway; 2 National University of Singapore, Singapore, Singapore; 3 Faculty of Science, Charles University in Prague, Prague, Czech Republic; 4 School of Computer Science and Engineering, Nanyang Technological University, Singapore, Singapore

## Abstract

Localization microscopy and multiple signal classification algorithm use temporal stack of image frames of sparse emissions from fluorophores to provide super-resolution images. Localization microscopy localizes emissions in each image independently and later collates the localizations in all the frames, giving same weight to each frame irrespective of its signal-to-noise ratio. This results in a bias towards frames with low signal-to-noise ratio and causes cluttered background in the super-resolved image. User-defined heuristic computational filters are employed to remove a set of localizations in an attempt to overcome this bias. Multiple signal classification performs eigen-decomposition of the entire stack, irrespective of the relative signal-to-noise ratios of the frames, and uses a threshold to classify eigenimages into signal and null subspaces. This results in under-representation of frames with low signal-to-noise ratio in the signal space and over-representation in the null space. Thus, multiple signal classification algorithms is biased against frames with low signal-to-noise ratio resulting into suppression of the corresponding fluorophores. This paper presents techniques to automatically debias localization microscopy and multiple signal classification algorithm of these biases without compromising their resolution and without employing heuristics, user-defined criteria. The effect of debiasing is demonstrated through five datasets of invitro and fixed cell samples.

## Introduction

Localization microscopy (LM) is an umbrella term referring to those super-resolution methods in fluorescence microscopy that exploit sparse spatio-temporal emissions of fluorophores (referred to as emitters for simplicity). The exploitation occurs in the form of localizing only a few optically separable emitters in each frame and performing such localizations for several frames, each with independent and sparse set of emitters. Localization of emitters is performed by fitting an estimated point spread function (PSF) to each intensity blob, which is potentially an image of an emitter, in each frame. Often, two-dimensional Gaussian function is used as an estimate of the PSF. Several techniques such as STochastic Optical Reconstruction Microscopy (STORM)^1^, Photo-Activated Localization Microscopy (PALM)^2,3^, Point Accumulation for Imaging in Nanoscale Topography (PAINT)^4^, Spectral Precision Distance Microscopy (SPDM)^5^, and their variants fall under LM. The individual implementations of LM vary in one or more of the following respects^6^: (a) the mechanism of inducing spatio-temporal sparsity of emissions; (b) the localization technique, which is generally based on either maximum likelihood estimation or least squares error minimization; (c) segmentation of regions of interest in a frame that potentially represent images of emitters; (d) heuristic filtering and clustering of localized emitters for constructing the LM image.

Recently proposed MUltiple SIgnal Classification ALgorithm (MUSICAL)^7^ demonstrates state-of-the-art super resolution of about 30 nm (two-point resolution, reported in^7^). It belongs to the family of methods that analyze the statistics of temporal fluctuation of intensity instead of performing localization in each frame independently such as done in LM. The analyzed temporal fluctuations are a result of blinking or bleaching dynamics of mutually independent emitters. There are few other methods in this family, summarized in^6^ and more recently in^8^. Among these methods, only MUSICAL and Bayesian analysis of blinking and bleaching^9^ reach resolution of less than 50 nm (2-point resolution) in their original form and unaided by hybridization with other methods. We note that the resolution values reproduced here are as reported in the main texts of the works^7,9^. Here, the effect of the chosen dye, labelling density, and experimental conditions are ignored, although these may have some effect on the achievable resolution. LM provides better two-point resolution than MUSICAL at ≈20 nm, although MUSICAL compares very well in terms of structural resolution, as seen in examples from EPFL dataset^10^ in^7^. The core advantage of MUSICAL over LM is the absence of the necessity of sparse emissions and strongly reduced requirements on the number of frames. We note that multi-fluorophore localization approaches, such as^9–14^, provide a slight advantage of density over single emitter localization approaches (only 5 to 8 fluorophores per square micron area per frame)^15^.

LM and MUSICAL not only differ in their requirements of spatio-temporal sparsity of emissions, they also differ in the treatment of the acquired data. LM processes one frame at a time such that spatial and temporal properties of data are dealt separately and sequentially. On the other hand, MUSICAL deals with spatio-temporal properties of data simultaneously by processing the entire image stack. As a consequence of these properties, LM and MUSICAL differ greatly in their treatment of temporal variations in the SNR in an image stack. LM counts all localizations with the same weight irrespective of their SNRs. Noisy localizations, which have a higher probability of being false localizations originating for example from background noise, are counted with the same weight as bright localizations, which almost certainly originate from the fluorescent labels. In this way, the potential false localizations often characterized with low SNR are inadequately over-represented in the final image. Most LM implementations suppress the contribution from false localizations by selecting only those localizations that meet certain criteria on intensity, spatial intensity distribution, or quality of its fit^16,17^. However, this frequently involves certain level of arbitrariness in the form of user-defined thresholds. On the other hand, MUSICAL under-represents the frames with low SNR by delegating a relatively large part of the intensity to the null space that represents noise. Thus, LM and MUSICAL both demonstrate a biased treatment of frames or regions with low SNR, although in opposite fashions. Here, we propose debiasing techniques to improve the performance of these methods in cases where their outputs suffer due to the bias. We show the effect of debiasing results using five experimental datasets, one dataset of *in-vitro* actin filaments, one dataset of *in-vitro* microtubules, and three datasets of microtubules in fixed cells.

## Materials and Methods

### *In-vitro* actin filaments

This data corresponds to *in-vitro* sample 1 of^7^ which is a stack of images of *in-vitro* actin filaments tagged with Phalloidin-Atto 565 dye in a STORM switching buffer. The stack has 10,000 images acquired at the rate of 200 frames per second in total internal reflection fluorescence microscope through a 100× magnifying oil lens of 1.49 numerical aperture. Emission wavelength of 593 nm was considered in the calculations. The readers are referred to the methods section of ^7^ for the sample preparation protocol and imaging details. The signal to background ratio for this data is ~2.

### *In-vitro* microtubules

This data corresponds to ‘Tubulins long sequence’ data from the single molecule localization microscopy (SMLM) dataset^18^. It is an image stack containing 15,000 images of a sample of microtubules. The image stack was acquired at 25 frames per second through an oil immersion lens of numerical aperture 1.3. The pixel size is specified as corresponding to 100 nm in the sample space. The emission wavelength is specified to be 690 nm. The readers are referred to^18^ for more details. The signal to background ratio for this data is ~3.

### Microtubules in fixed cells

Fixed U2OS cells with tubulin immunolabelled by Alexa 647 were imaged by Nikon Eclipse Ti inverted epifluorescence microscope with NSTORM module (Nikon, Japan). The sample was illuminated in widefield mode by a 640 nm laser line through a 100× magnifying oil lens of 1.49 numerical aperture (HP Apo TIRF, Nikon, Japan). Optionally, simultaneous illumination by a 405 nm laser line was used to further promote the switching of the fluorophore between its bright and dark states. Stacks of 10,000 images were captured by Hamamatsu Orca-flash 4.0 camera at 100 frames per second rate. The total magnification of the microscope was adjusted by additional lenses to correspond to 108 nm per pixel. The cells were kindly provided by Dr. Ivan Novotný (Institute of Molecular Genetics of ASCR). They were kept in phosphate buffer which was replaced with STORM switching buffer^7^ prior to imaging. The signal to background ratio for this data is in the range 3 to 7.

### Synthetic example

Synthetic example considers geometry of a fork. The fork has two prongs with an angle of 30° between them attached to a stem; each prong and the stem are 500 nm long and have randomly distributed 100 emitters, each. Blinking of emitters is simulated using Poisson blinking model, where the length of switching times *t*_on_ and *t*_off_ are computed using a Poisson distribution with the average on time ${\tau }_{{\rm{on}}}=5$ ms and the average off time ${\tau }_{{\rm{off}}}=95$ ms, respectively. The photon emission rate is assumed to be 10^5^ photons sec^−1^. For simulating blinking, we generate a random number for each active (unbleached emitter) in the range [0, 1] assuming uniform distribution and compared it with the function $\exp (-t/{\tau }_{{\rm{bleach}}})$ where *t* is the time elapsed since the beginning of data acquisition and ${\tau }_{{\rm{bleach}}}=540$ ms is the time constant of bleaching. If the random number is more than the value of this exponential function, it is considered bleached. This process is performed for each active emitter before simulating blinking for the next frame. An imaging system with numerical aperture 1.49 and magnification 1 and a camera of pixel size 100 nm are assumed. Image stack contains 10000 frames acquired at an image acquisition rate of 50 frames per second and has a signal to background ratio of 100.

### MUSICAL

Matlab implementation of MUSICAL^7^ was used for generating results of both the original and debiased versions. MUSICAL was performed using ${\sigma }_{0}=0.01{\sigma }_{1}$ and *α* = 4 for all the results where the entire image stack was used. Here, *σ*_1_ is the largest singular value of the image stack. For the MUSICAL images computed using subsets of the image stack, the values of *σ*_0_ were chosen according to the knee criterion discussed in the supplementary information of ^7^. Soft window of size 7 and a Gaussian soft window function with the root mean square width of 3 were used for all MUSICAL results. The formation of the color map and the color values are derived directly from the supplementary information of ^7^. The debiasing techniques for MUSICAL are applied on the image stacks before executing MUSICAL.

### LM

rainSTORM implementation^19^ of STORM was used as the representative of LM. For all the results, approximate radius of 1.5 was provided as input. A maximum of 10 iterations were used for the least squares fitting of each emitter. The relative tolerance of fitting was specified to be 0.001. The formation of the color map and the color values are derived directly from the supplementary information of ^7^. For the data of microtubules in fixed cells, we generated LM results using both rainSTORM and NSTORM. We have purposely used the configurations which allow a parametric match between the rainSTORM implementation and the NSTORM implementation. Specifically, we used a thorough search segmentation for regions of interest in both the implementations and the same value of radii of PSF. We note that the default setting of NSTORM does not use a thorough search and thus provides better reconstruction with lesser artifacts. However, the specific details regarding such selectivity are unavailable due to proprietary nature of NSTORM. The additional default computational filters used in NSTORM are toggled off before generating the NSTORM results. If such filters are used, they are specifically reported with the corresponding figures.

### Source code availability

The source codes for all the debiasing techniques shall be made available on https://sites.google.com/site/uthkrishth/musical.

### Data Availability

The codes used for generating the data in this manuscript shall be made available at (https://sites.google.com/site/uthkrishth/musical) after the acceptance of the manuscript.

## Results and Discussions

### The biased role of the signal to noise ratio (SNR) in LM and MUSICAL

In LM, fluorophores blink so sparsely over time that the density of fluorophores emitting at a given time is small. Consequently, the likelihood of the emitters being optically separable is high. Subject to the number of photons emitted, shot noise, and electronic noise, the emitters can be localized using least squares error minimization or maximum likelihood estimation (MLE). The concept applies to all forms of localization microscopy and an excellent review can be found in^6^. We note that sparse temporal blinking or selective activation of fluorophores can be induced through the use of photo-activable or photo-switchable dyes and proteins, reductive oxygen-deficient imaging medium which regulates quenching and blinking in dyes, or use of high intensity laser for excitation or activation^20^.

MUSICAL computes eigenimages of the image stack and uses the singular values associated with them to classify them as belonging to signal (*S*) subspace or the null (*N*) subspace of the measurements. We refer the readers interested in the details of eigenimages, signal subspace and null subspace to the supplementary material of^7^. We briefly clarify here that eigenimages are the eigenvectors obtained after eigenvalue decomposition of the data matrix in which rows correspond to spatial information (pixels) in the image stack and columns correspond to temporal information (frames). Eigenimages are slightly different from the principal components in the sense that principal components are computed using the covariance matrix of the data matrix mentioned above. Thus, the mean component (i.e. mean image of the image stack^8^) is retained in eigenimages but absent in principal components. Then, it computes the projections *d*_*s*_ and *d*_*N*_ of the point spread function at a test point on the signal and null subspaces. These projections indicate the extent to which the measured intensity corresponds to noise and to the photon emissions from the emitters. Lastly, it computes ${({d}_{S}/{d}_{N})}^{\alpha }$, a function that rewards the contribution to signal and penalizes the contribution to noise. A key parameter of MUSICAL is the threshold *σ*_0_ on singular values that divides the measurement space into the signal and the null subspaces.

While the modus operandi of LM and MUSICAL appear different, they are tied together through the conceptual exploitation of noise in processing the raw data. The role of noise in LM is less apparent than in MUSICAL where it enters explicitly through *d*_*N*_. Consider the least squares error minimization approach of LM^21^. When fitting a two-dimensional Gaussian to noise, either the fit is very poor, the fact being reflected in the least squares error, or the fitted Gaussian is wide and inconsistent with the PSF of the system. LM rejects these cases since they do not meet internal criteria for localizations. (see^19^ for example). Similar considerations apply for MLE approach^22,23^.

**Figure 1:**
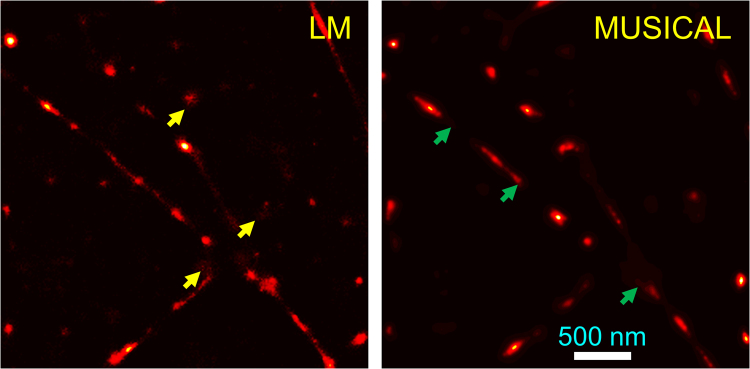
Examples of bias in LM (left image, yellow arrows) and MUSICAL (right image, green arrows).

LM and MUSICAL deviate from each other in their treatment of the temporal characteristics of SNR, and it is arguable that neither is optimal in their conventional form, as we explain below. LM performs localizations in each frame independent of the other frames and retains only the localized coordinates for forming the final LM image. In essence, this flattens the image stack temporally, where each frame is given equal weight irrespective of the SNR of the emitting fluorophores in the image stack. However, the measured fluorescent intensities exhibit temporal variations in response to natural photokinetic phenomena of fluorophores such as bleaching, changes in chemical composition of the imaging buffer during imaging^24^, or experimental intervention in photokinetics of fluorophores such as through activation of secondary lasers or increasing the excitation power. Examples to illustrate the temporal variations are presented in Supplementary Note 1. The consequence of the temporal variations is that the SNR of the frames may vary depending upon the fluorescence intensity, resulting in localizations of poorer quality (lower value of maximum likelihood or higher least squares error) for frames with low SNR. The poorer quality in turn may result in increased chances of imprecise, inaccurate, or false localizations. This is often manifest as cluttered background or random non-structural patches in the background. An example is shown in Fig. 1. In this example, the yellow arrows in LM image show the clutter in background due to localizations performed on blobs in frames with poor SNR.

MUSICAL computes eigenimages and corresponding singular values from all the frames in the image stack. The frames with high SNR make larger contribution to the eigenimages with large singular values. Thus, whatever threshold is used for assigning signal and null subspaces, the signal subspace is biased towards the frames with high SNR. The risk of choosing smaller threshold is that the noise in the frames with low SNR contributes to the signal subspace (although under-represented) while the risk of using large threshold is that the signal in the frames with low SNR contributes negligibly to the signal subspace. Consequently, the MUSICAL reconstruction is noisy in the first case and it misses features with poor emissions in the second case. Examples of missed features due to poor emissions is shown in Fig. 1 using green arrows. Further illustrations with more details about the biases of LM and MUSICAL appear later in results for *in-vitro* actin filaments.

Through the above discussion, we highlight that in their current forms, LM and MUSICAL do not deal adequately with spatio-temporal variations of SNR. While LM may be prone to over-representing low SNR regions, MUSICAL under-represents the low SNR frames. In the next section, we present techniques to address this biased treatment by adequately compensating for spatio-temporal SNR variations encountered commonly in LM and MUSICAL data. Most importantly, the proposed techniques are very generic and fully automatic without the need for any user-defined parameters.

### Debiasing LM

**Figure 2:**
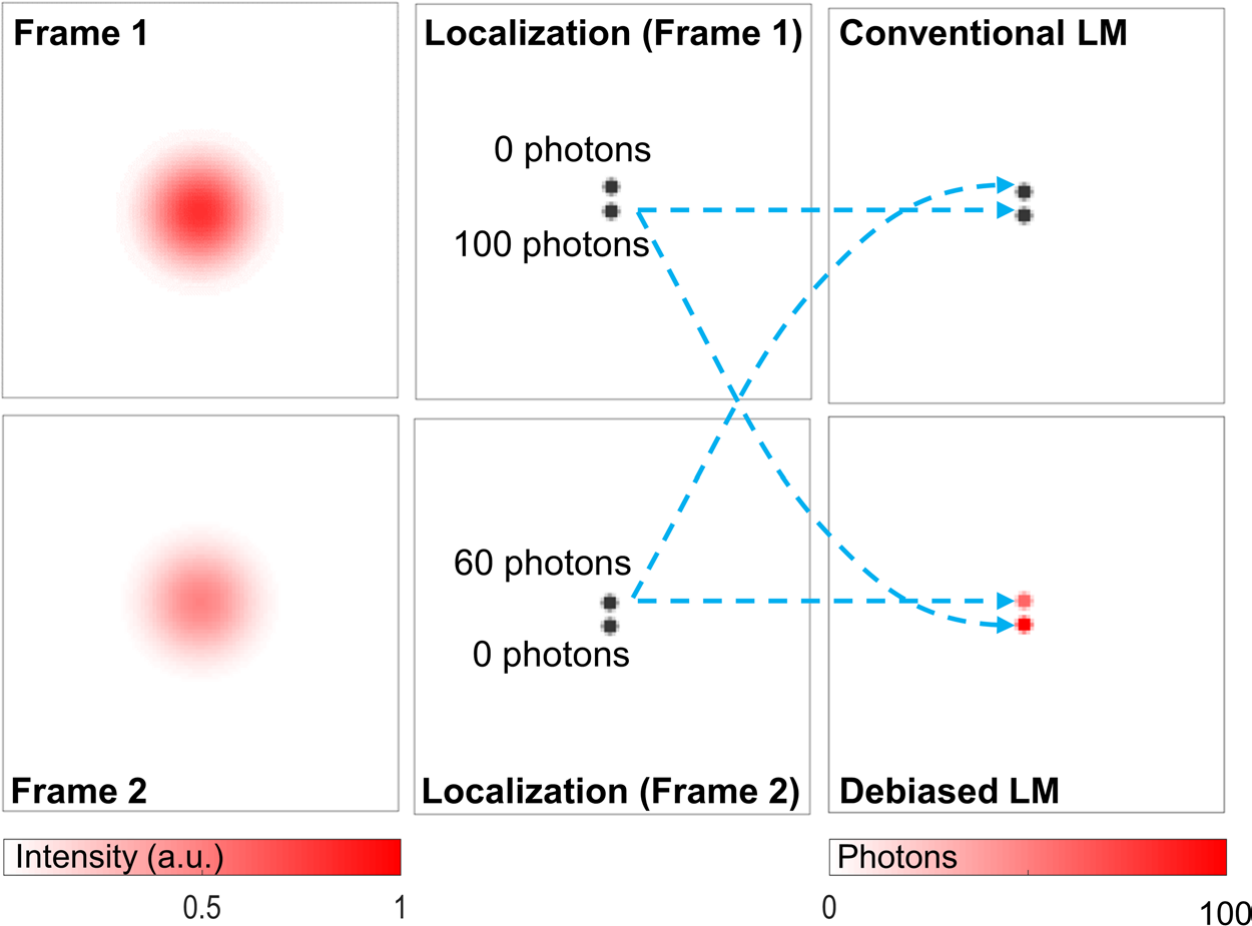
Illustration of the debiasing technique for localization microscopy through an example of two emitters with distance between them being a small fraction of the Abbe’s diffraction limit.

We do not alter the process and results of localization in each frame but modify the construction of LM image from the localization results. This is done through inclusion of temporal characteristics of intensities. The construction of LM image is mathematically expressed as:

1 $$s(x,y)=\sum _{k}{a}_{k}(x,y)$$

where

2 $${a}_{k}(x,y)=\{\begin{array}{ll}{b}_{k}(x,y) & {\rm{if}}\,\exists \,{\rm{localization}}\,{\rm{at}}\,(x,y)\,{\rm{in}}\,{\rm{the}}\,k{\rm{th}}\,{\rm{frame}}\\ 0 & {\rm{otherwise}}\end{array}$$

where *x* and *y* represent the coordinates in the local lateral plane and *k* represents the frame number (i.e., the temporal coordinate). A discretized LM image is constructed as follows:

3 $$S({x}_{p},{y}_{p})={\int }_{(x,y)\in {{\rm{\Omega }}}_{p}}s(x,y){\rm{d}}x{\rm{d}}y$$

where *x*_*p*_ and *y*_*p*_ are the center coordinates of the *p* th pixel and ${{\rm{\Omega }}}_{p}$ is the spatial region spanned by the *p* th pixel. We note that advanced clustering approaches^25^ such as k-means clustering and Voronoi tessellations^26^ are being adopted recently. In such approaches, clustering equation can be represented as summation over a cluster rather than integration over spatial coordinates shown in eq. (3). Besides this, the general framework remains the same and the remainder of discussion remains applicable. In the conventional construction of LM, ${b}_{k}(x,y)=1$ is used. Or if heuristic filters are employed, ${b}_{k}(x,y)$ takes binary values; if the parameters of fitted PSF satisfy the filter criterion, ${b}_{k}(x,y)$ is set as 1, and 0 otherwise^16,17^. This implies a strict retain-or-reject approach. We discuss examples of such filters while discussing the LM results for microtubules in live cells. We modify the function ${b}_{k}(x,y)$ for debiasing LM as follows:

4 $${b}_{k}(x,y)={\psi }_{k}(x,y)$$

where ${\psi }_{k}(x,y)$ is the estimated number of photons emitted by the localized molecule. In this technique, we allow each localization to represent itself in terms of its estimated photon emissions. Since the estimated number of photons is directly related to the intensity and the SNR of the pixels participating in localization, both the SNR of the frame and the local spatial characteristics participate in the construction of the LM image in this technique. The debiasing technique is illustrated in Fig. 2.

We investigated two additional techniques for debiasing LM in Supplementary Note 3 in order to assess the possibility of frame-wise weighing instead of localization-wise weighing described above. For convenience, we refer to the above presented technique as technique 1. Technique 2 debiases LM by weighing the localizations in a frame with the frame’s average intensity. Technique 3 debiases LM by weighing the localizations in a frame with the average intensity of the foreground only. The technique to extract the foreground is discussed in Supplementary Note 2. Lastly, we consider one more debiasing technique that incorporates the information of local signal to background ratio through localization accuracy of each localization^21^. We refer to this as Technique 4. We discuss the techniques 2 to 4 and their comparison with technique 1 in Supplementary Note 3.

## Debiasing MUSICAL

The details of MUSICAL can be found in^7^. Here, we assess the contribution of a frame to signal and null subspaces as a consequence of the threshold *σ*_0_. Let the image in the *k* th frame be denoted as **I**_*k*_. Let weighted image frames be collected in an image stack **I** such that each column in the matrix **I** is ${w}_{k}{{\bf{I}}}_{k}$. For the conventional form of MUSICAL, *w*_*k*_ = 1 is used.

Let the singular values of the image stack **I** sorted in descending order be denoted as ${\sigma }_{1}$, ${\sigma }_{2}$, $\ldots$, ${\sigma }_{i}$, $\ldots$, ${\sigma }_{\min N,K}$, where *N* and *K* are the number of pixels in the selected spatial window and the number of frames in the image stack. It was shown in^8^ that the eigenimage corresponding to ${\sigma }_{1}$ denotes the mean image of the image stack. We define

5 $$\nu =\sqrt{\sum _{{\sigma }_{i}\le {\sigma }_{0}}{\sigma }_{i}^{2}}$$

**Figure 3:**
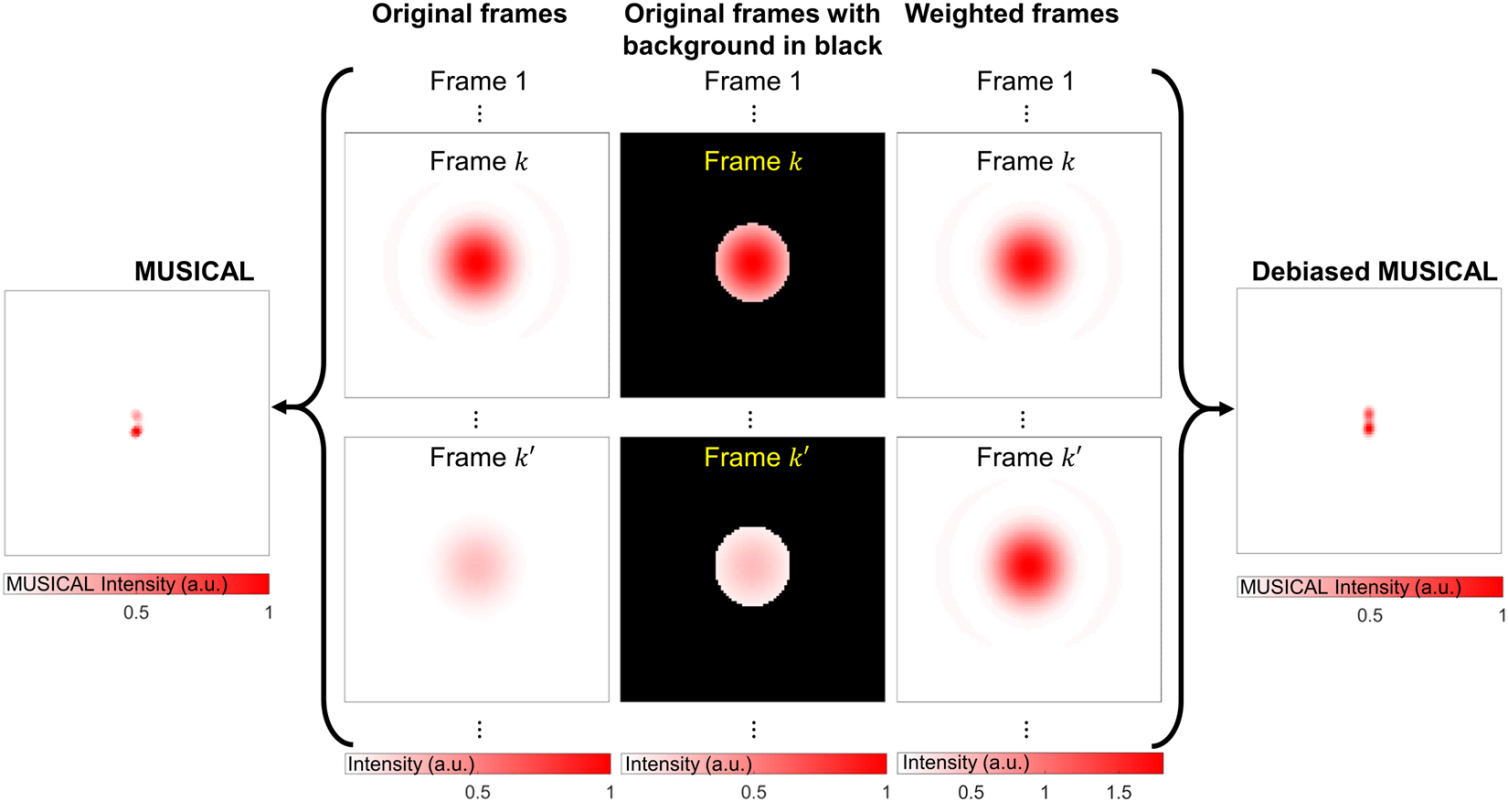
Illustration of the debiasing technique for MUSICAL. The example is the same as Fig. 2, however more than two frames are needed in MUSICAL for imaging two independent emitters^7^. In the original MUSICAL, the frames are used directly in the MUSICAL algorithm. In the debiased MUSICAL, we determine the intensity of the foreground in each frame and weigh the frame such that all the frames have similar foreground intensity. The weighed frames are then used in MUSICAL to reconstruct the debiased MUSICAL image.

Then, ${\sigma }_{1}/\nu$ denotes the ratio of the mean intensity to the portion of image stack relegated to the null subspace. If all the frames are given the same weights, i.e. ${w}_{k}=1$ is used, then the portion of the frames which are relegated to the null space is constant irrespective of the frames’ intensities. While this fares well for Gaussian noise, it is unsuitable for microscopy images which have Poisson noise that varies with the signal strength. The debiasing technique proposed for MUSICAL uses *w*_*k*_ defined as follows:

6 $${w}_{k}={(\frac{{\sum }_{\forall r^{\prime} \in F}I(r^{\prime} )}{{N}_{r^{\prime} }})}^{-1}$$

where $r^{\prime}$ denotes a pixel, *F* denotes the foreground in a frame, and ${N}_{r^{\prime} }$ is the total number of foreground pixels. The debiasing technique is illustrated in Fig. 3. The image stack formed with weighted image frames is then used as input for the MUSICAL algorithm.

Our foreground detection technique is presented in Supplementary Note 2.We highlight that this foreground detection technique is different from the conventional foreground detection by thresholding the intensities which exploit the assumption of bimodal distribution of histogram of intensities (HoI) in an image^27^. Our approach models the histogram of the logarithm of the HoI in an image frame as a bimodal distribution for extracting the foreground. Other approaches for foreground detection may also be suitable^28^.

In the proposed debiasing technique, all the pixels in a frame are weighted by the same value of *w*_*k*_. Thus the signal to background ratio of the frame remains unchanged and no local spatial variations are introduced in the frame. Also, note that the weighing does not alter the signal to noise ratio of any pixel in the frame, but makes the intensities of the foreground comparable across the frames.

For convenience, we refer to the above proposed technique as technique 1 in Supplementary Note 4 for debiasing MUSICAL. We investigated two additional techniques for debiasing MUSICAL, which are presented and compared with technique 1 in Supplementary Note 4. Technique 2 uses the average intensity of the frame to debias MUSICAL while technique 3 uses the standard deviation of the intensities in the frame to debias MUSICAL.

### *In-vitro* actin filaments

**Figure 4:**
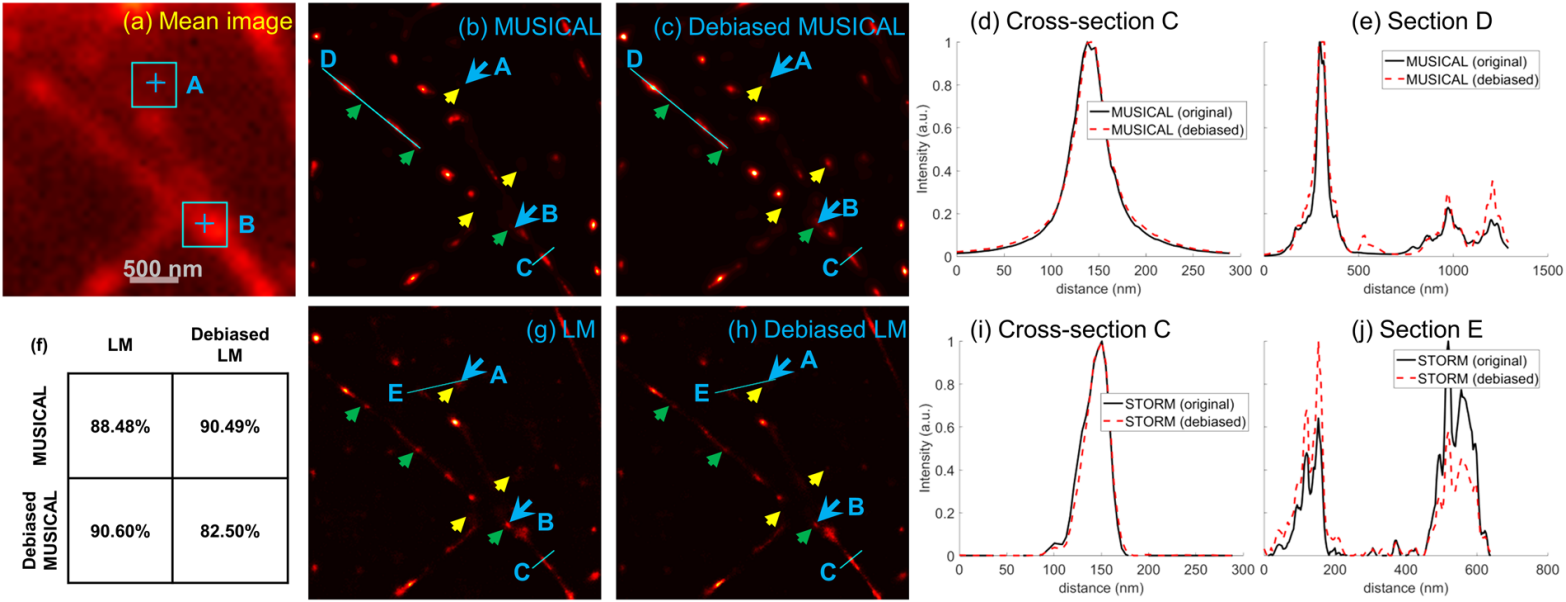
Results of original and debiased forms of LM and MUSICAL for *in-vitro* actin filaments are shown here. The table shows SSIM values of corresponding pairs of images. Small yellow and green arrows are replicated from Fig. 1. The blue colored ‘+’ and squares in the mean image, and corresponding large blue arrows, indicate points A and B used in Figs 5 and 6.

This data corresponds to the *in-vitro* sample 1 of^7^. This data was used to generate the LM and MUSICAL results in Fig. 1. The mean image of the image stack, the results of the original form of LM and MUSICAL, and the results of debiased LM and debiased MUSICAL are given in Fig. 4. The yellow and green arrows from Fig. 1 are replicated here for qualitative comparison of the effect of debiasing. Consider the intensities of original MUSICAL and debiased MUSICAL at section D plotted in Fig. 4(e). The capability of debiased MUSICAL in enhancing the features corresponding to low SNR is evident. Similarly, debiasing LM has resulted in suppression of the LM intensity as the locations of yellow arrows. This is further validated through section E shown in Fig. 4(g,h). Intensities of original LM and debiased LM for this section are plotted in Fig. 4(j). It is seen that the debiased LM reduces the intensity of the blob corresponding to low SNR. Yet, the spreads of MUSICAL and LM do not degrade due to debiasing, as seen through the plots of intensities at cross-section C, plotted in Fig. 4(d,i). It is noted in comparison of sections C–E that the structural resolution of these methods is not compromised.

Further, qualitative inspection reveals that the debiased MUSICAL image has several features that are missing in the original MUSICAL image but present in the original LM image. On the other hand, the debiased LM image has several qualitative similarities with the original MUSICAL image.

Structural SIMilarity (SSIM) index is used to quantify the perceptual similarity between two images^29^ in terms of their structures. Since the super-resolution images generated by different approaches are not comparable directly in terms of the pixel-by-pixel differences, SSIM is better suited than other image comparison metrics such as mean square error or peak signal-to-noise-ratio for comparing super-resolution images of different algorithms. The SSIM values of various pairs of images is also given in Fig. 4. It is seen that the SSIM values for the pair ‘original MUSICAL - debiased LM’ and the pair’ debiased MUSICAL - original LM’ are better than the pair ‘original MUSICAL - original LM’ by >2%. This indicates that the debiasing techniques for both techniques indeed compensate for the biased representation of frames with low SNR.

**Figure 5:**
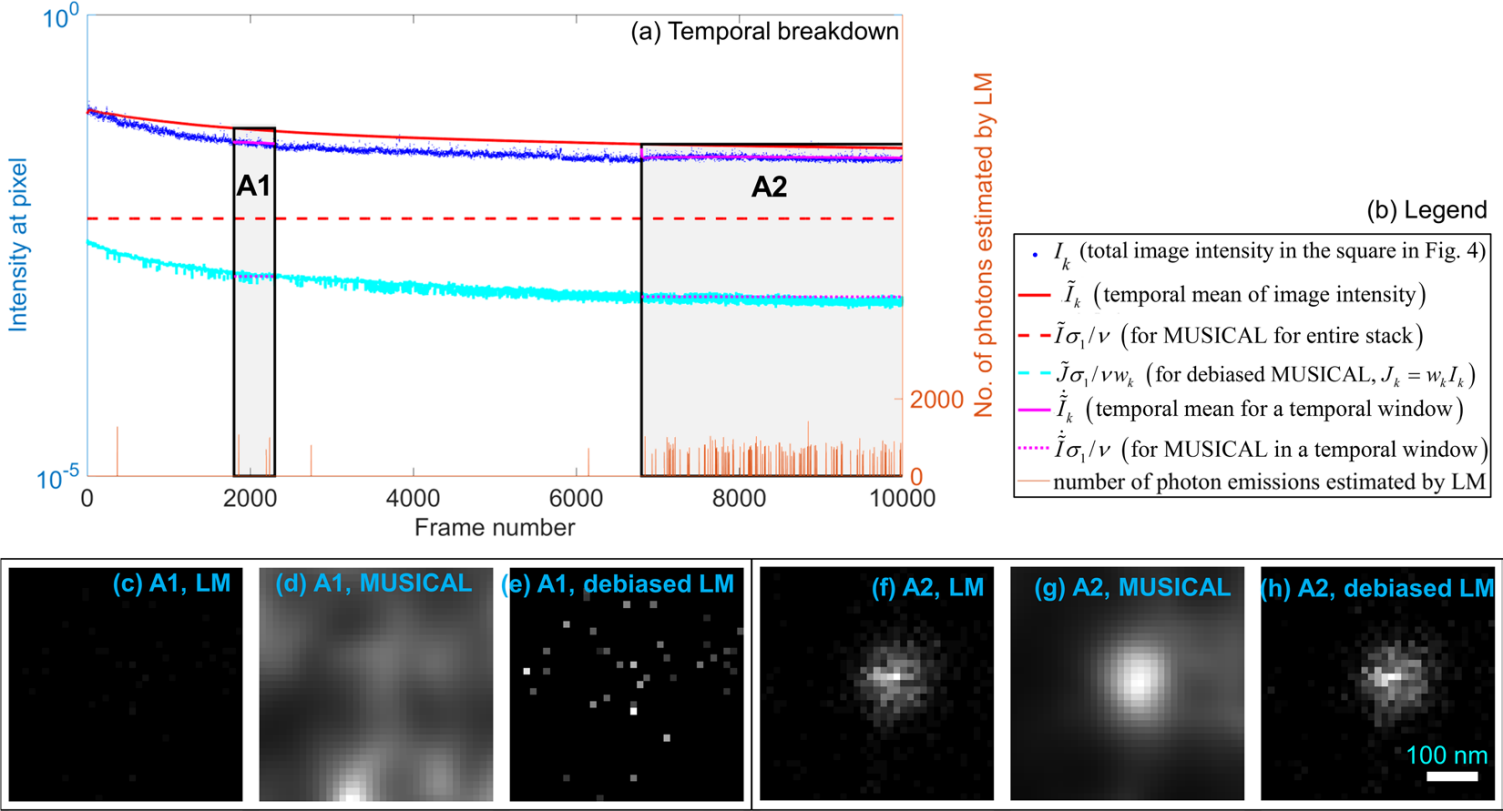
Temporal breakdown of LM and MUSICAL for the square region (size 455 nm) around point A shown in Fig. 4(a). The brightness of (**c**,**f**) represents the number of localizations and have the same scale, (**d**,**g**) represents the MUSICAL intensity and have the same scale, and (**e**,**h**) represents the estimated number of photons emitted from the localized emitters and have the same scale. The pixel size in the reconstructed images (**c**–**h**) is 13 nm.

**Figure 6:**
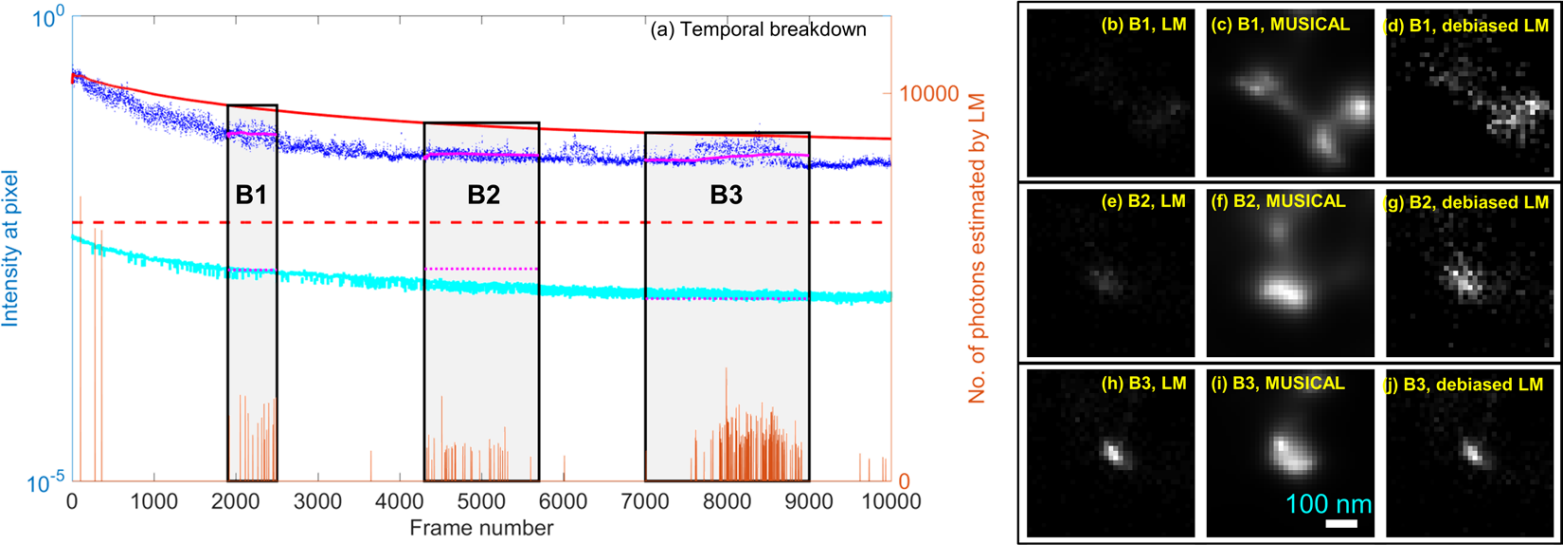
Temporal breakdown of LM and MUSICAL for square region around point B shown in Fig. 4(a). The legend for subfigure (**a**) is the same as Fig. 5(b). The brightness of (**b**,**e**,**h**) represents the number of localizations and have the same scale, (**c**,**f**,**i**) represents the MUSICAL intensity and have the same scale, and (**d**,**g**,**j**) represents the estimated number of photons emitted from the localized emitters and have the same scale. The pixel size in the reconstructed images (**c**–**h**) is 13 nm.

We consider points A and B shown using blue colored ‘+’ and large blue colored arrows in Fig. 4 for an insight into the debiasing techniques. The detailed results for point A and point B are shown in Figs 5 and 6, respectively. The blue colored squares around the ‘+’ marks in the mean image indicate the window used for computing MUSICAL results in Figs 5 and 6.

The plots in Figs 5(a) and 6(a) provide a temporal breakdown of various intensities, temporal windows being investigated, and localizations. The blue colored dots show the sum of intensities, represented for convenience as *I*_*k*_, in the square spatial windows shown in Fig. 4. The red colored solid line shows ${\tilde{I}}_{k}$, the mean image considering frames $[1,k]$. The red dashed line indicates the effective portion of intensity relegated to null subspace, given by $\tilde{I}{\sigma }_{1}/\nu$, when the entire image stack is used for MUSICAL. It indicates that a large portion of the intensity is relegated to null subpsace in the later frames with lower intensity and thus poorer SNR. Relatively smaller portion of the intensity is relegated to the null subspace in the initial frames with higher intensity and thus better SNR.

Now, we consider temporal sub-stacks of the image stack, such as A1 and A2 for point A and B1–B3 for point B. For each sub-stack, magenta colored solid line is equivalent to the red solid line, i.e. ${\tilde{I}}_{k}$, however for this sub-stack only. Similarly, magenta colored dashed line is equivalent to red colored dashed line, however for this sub-stack only. Consider image stack **J** containing weighted image frames ${J}_{k}={w}_{k}{I}_{k}$, where *w*_*k*_ from eq. (6) is used for debiasing MUSICAL. Then $\tilde{J}{\sigma }_{1}/\nu$ is the threshold for the weighted image stack, where ${\sigma }_{1}$ and *v* are calculated for **J** and $\tilde{J}$ is the mean image of **J**. Then in the context of the original frames *I*_*k*_, the contribution to null subspace is given by $\tilde{J}{\sigma }_{1}/\nu {w}_{k}$. This is shown using a cyan line. Interestingly, the cyan line is quite close to the magenta colored dashed lines which represent the contribution to the null subspace of the corresponding sub-stacks. This shows that the threshold represented by cyan line is more effective in relegating suitable portion of the intensity to the null subspace.

Lastly, we use LM to determine the frames in which there is a localization in a small region (diameter 16 nm) centered at the point A or B. The estimated number of photons computed by LM in these frames is denoted by the amplitudes of the orange spikes in these plots. Original form of LM simply uses the number of frames in which there were localizations (i.e. number of spikes). Debiased LM on the other hand uses the amplitudes of these spikes as well.

Now, we discuss the results of sub-stacks A1 and A2. It is evident in Fig. 5(a) that the localizations from LM are concentrated mainly in the trailing frames in sub-stack A2. Their localization strengths are small in comparison to some localizations in Fig. 6(a). Thus, though there are localizations in these last frames, they may represent late emitting emitters or simply noise.

Results of LM and MUSICAL are similar for sub-stack A2. However, MUSICAL result for the entire stack (Fig. 4) shows negligibly small MUSICAL intensity at point A. This is because MUSICAL for the entire stack is dominated by a threshold significantly higher than the threshold for sub-stack A2 alone. This is evident in the red dashed line and the magenta dashed line in sub-stack A2 in Fig. 5(a).

On the other hand, debiased MUSICAL shows a spot at the point A in Fig. 4 since the debiasing helps the MUSICAL threshold to adapt to these later frames as shown through cyan line in Fig. 5(a). For the sub-stack A2, the results of debiased LM and original LM appear similar since the estimated number of photons is relatively the same for all these frames. However, the total number of photons estimated at point A is much smaller than point B. Thus, the spot at point A clearly seen in original LM is significantly suppressed in debiased LM in Fig. 4.

Sub-stack A1 is a trivial sub-stack with hardly any localizations. This is possibly a situation of high background due to other emitters in vicinity, but no local emissions in this temporal window. With an appropriate threshold for this sub-stack, MUSICAL shows a relatively flat reconstruction analogous to background. However, original LM is the most effective since there are almost no localizations. Although debiased LM generates spurious spread of intensity comparable to the debiased LM for A2, it is still insignificant in comparison to debiased LM for point B.

It is seen in Fig. 6 that the MUSICAL images for sub-stacks B1–B3 match very well with the debiased LM for the respective sub-stacks. This example illustrates how debiasing LM and MUSICAL may be able to bring them to a similar treatment of SNR, in which the frames with low SNR are neither over-represented not under-represented. Interestingly, MUSICAL images of none of these sub-stacks are close to the MUSICAL image or debiased MUSICAL image for the entire image stack shown in Fig. 4. The same applies for LM and debiased LM as well. This is because the fluorophores that emit in these sub-stacks are only a subset of all the fluorophores.

### *In-vitro* microtubules

**Figure 7:**
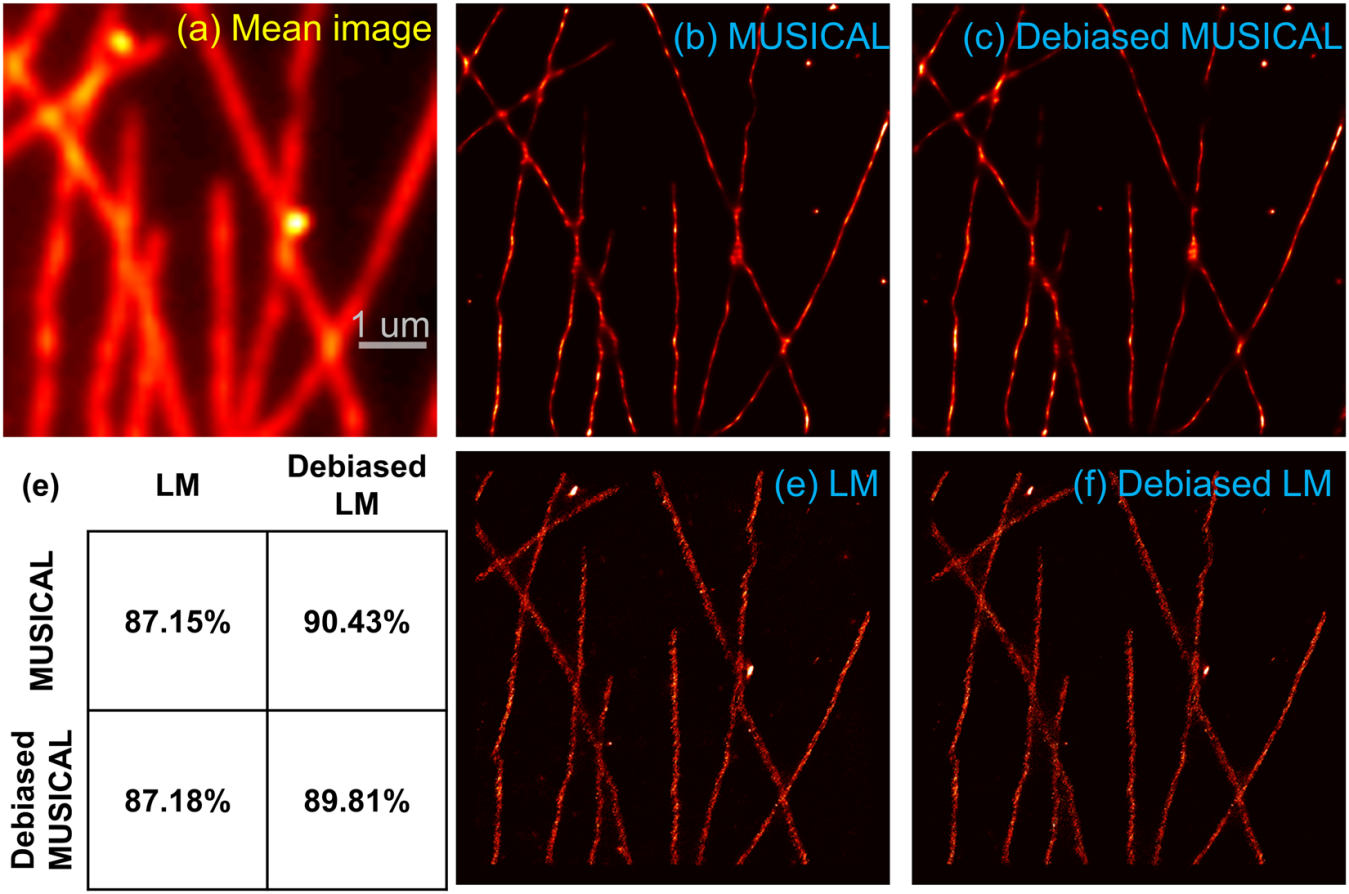
Results of original and debiased forms of LM and MUSICAL for *in-vitro* microtubules are shown here. The table shows SSIM values of corresponding pairs of images.

**Figure 8:**
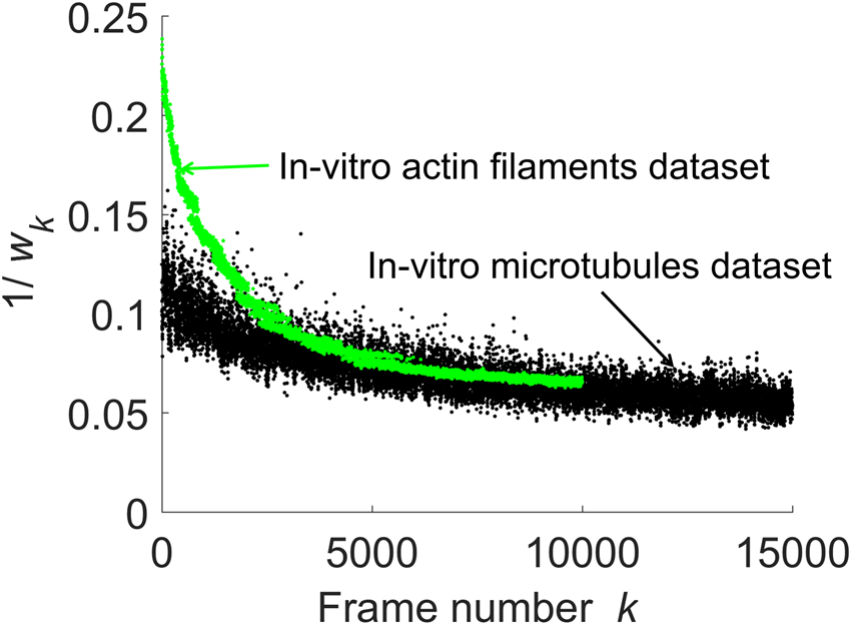
The values of $1/{w}_{k}$ computed using eq. (6) for the two datasets considered in this paper are shown here. *In-vitro* microtubules dataset is characterized by high sparsity of emissions, which results in relatively flat trend of $1/{w}_{k}$. Debiasing MUSICAL for such case is trivial, as noted in Fig. 7. Large variation in the range of $1/{w}_{k}$ for the *in-vitro* actin filaments indicates the need of debiasing MUSICAL for this data.

This data corresponds to the ‘Tubulins long sequence’ data from the SMLM dataset^18^, which is characterized by extremely sparse emissions captured over 15,000 frames. The mean image of the image stack, the results of the original form of LM and MUSICAL, and the results of debiased LM and debiased MUSICAL are given in Fig. 7. For this example, the effect of debiasing LM is prominent. The SSIM for the pair ‘original MUSICAL - debiased LM’ is >3% better than the SSIM for the pair ‘original MUSICAL - original LM’. However, debiasing of MUSICAL is ineffective. This is the consequence of very sparse emissions. Because of the high sparsity of emissions, the effective size (number of pixels) and mean intensity of the foreground does not change significantly over the frames. Thus, $1/{w}_{k}$ is flatter in comparison with the previous data. This is shown in Fig. 8. This indicates that debiasing of MUSICAL is helpful in conditions not characterized by extremely sparse emissions, in which case it is trivial. On the other hand, debiasing of LM can be effective in most situations where LM is applicable.

### Simulated example of fork

**Figure 9:**
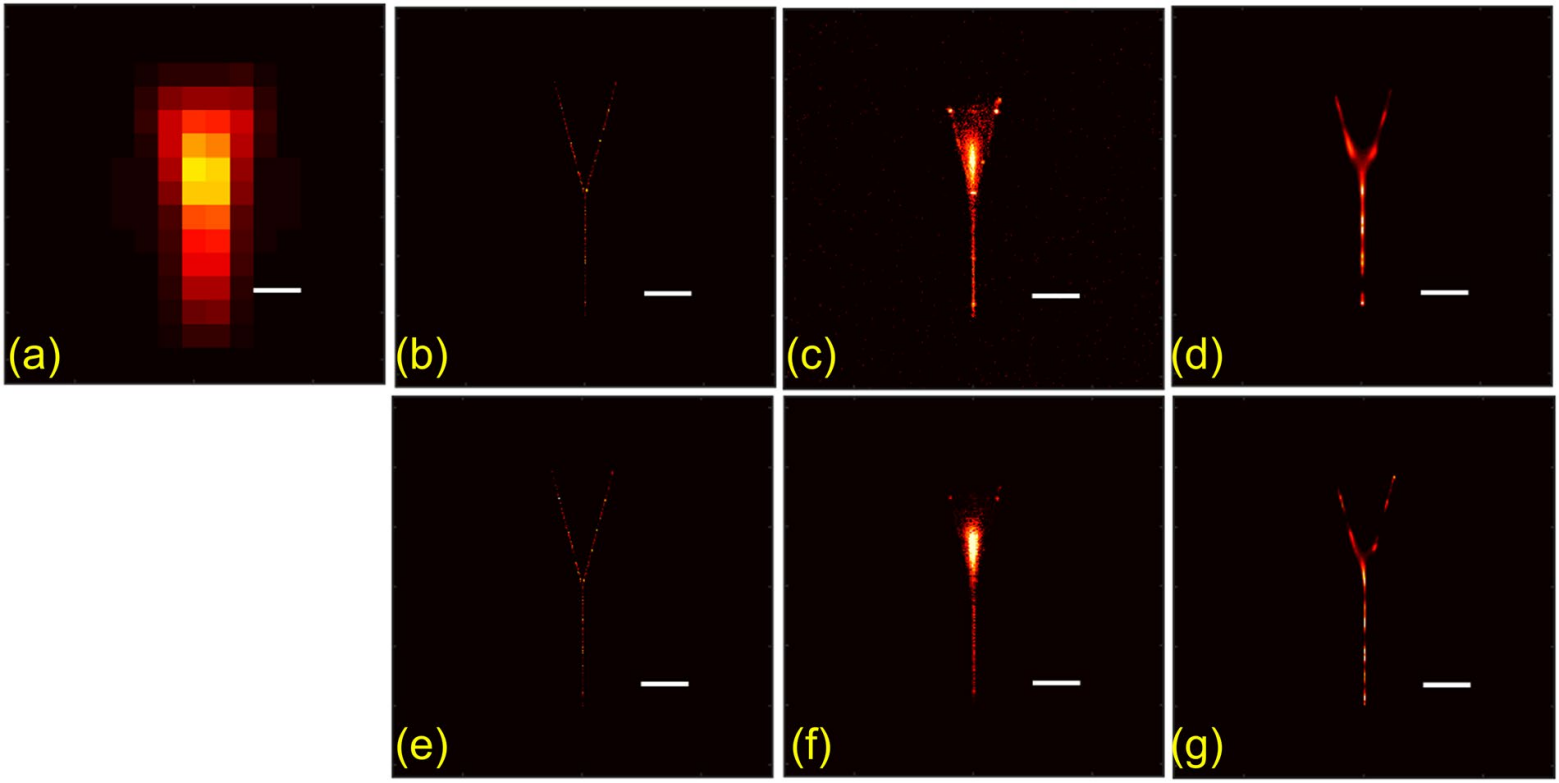
Synthetic example of fork is used to illustrate the effect of debiasing LM and MUSICAL in the regions of sparse emitter distribution (stem and the open ends of the prongs) and dense emitter distribution (the junction of the prongs). (**a**) Shows the mean image. (**b**) Shows the map of total number of events of actual emissions (ground truth for original LM). (**e**) Shows the map of total number of actual photon emissions (ground truth for debiased LM). (**c**,**f**) Are the results of original and debiased LM. (**d**,**g**) Are the results of original and debiased MUSICAL. Scale bar: 200 nm.

In order to further illustrate the points discussed before, we consider a synthetic example of a fork as shown in Fig. 9. The details of simulations are provided in the methods. This example illustrates high density of emitters at the junction of the prongs whereas sparsity along the open ends of the prongs and the length of the stem. The positive effect of debiasing LM is clearly evident in the stem and in the suppression of false localizations in the background. However, the mislocalizations get emphasized in debiased LM at the junction of the two prongs. On the other hand, the positive effect of debiasing MUSICAL is more pronounced at the junction of the prongs.

**Figure 10:**
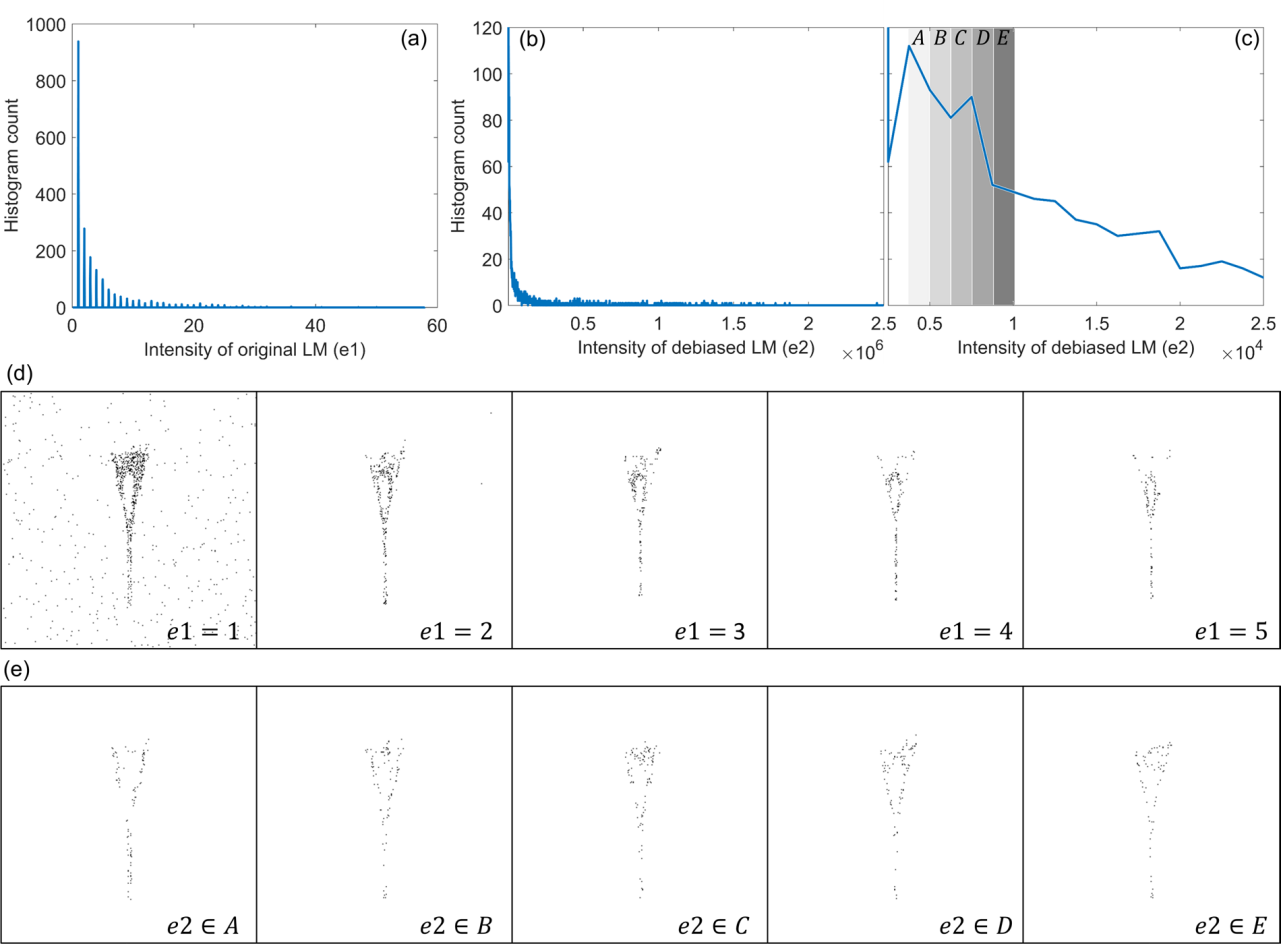
Synthetic example of fork is used to illustrate an advantage of debiasing LM. (**a**) And (**b**) Show histogram of intensities $s(x,y)$ for original and debiased LM, briefly denoted as *e*1 and *e*2, respectively. The lowest intensity bin, corresponding to background, has not been shown here. (**c**) Shows a zoom-in of (**b**) for $e2 < 2.5\times {10}^{4}$ and marks 5 sets *A-E* for values of *e*^2^. (**d**) Shows binary segmentations for the discrete values of *e*_1_, i.e. intensity $s(x,y)$ of the original LM. Thus, $e1=1$ implies the binary segmentations corresponding to only one localization in each pixel in the LM image. (**e**) Shows binary segmentations for the ranges *A–E* of $e2$, i.e. intensity $s(x,y)$ of the debiased LM, shown in (**c**). Thus, $e2\in A$ shows the binary segmentations of the pixels in the LM image with total number of photons in the range $A$ shown in (**c**). Details about histograms are in Supplementary Note 5.

We use this synthetic example to illustrate an advantage of the proposed debiasing of LM. The histograms of the intensities of original and debiased LM are shown in Fig. 10(a,b). Since the original LM counts the number of localizations, the histogram of original LM is discrete. On the other hand, the range of photon counts is very large to provide a practically smooth distribution. Here, we have used 2000 histogram bins in Fig. 10(b). This provides a unique opportunity for flexibly segmenting the debiased LM images. As an example, we show segmentations using discrete values of original LM image of fork in Fig. 10(d) and different ranges of debiased LM image in Fig. 10(e). It is seen that none of the five segmentations in Fig. 10(d) are able to isolate the features of fork from the incorrect localizations. On the other hand, segmentations corresponding to ranges *A* and *B* in Fig. 10(e) are more effective in suppressing the inaccurate localizations. Practically speaking, if the expected number of photons from a single emitter and the density of emitters can be estimated through information about the dye or post-processing, reliable segmentations of the structures can be derived.

### Microtubules in fixed-cells, debiasing of two different implementations of LM

This section considers three datasets of microtubules in fixed cells. We use these examples to show the effect of debiasing LM on different implementations of LM and how debiasing may be used for bridging the gap between two different implementations and emphasizing the features characterized by better signal to noise ratio, irrespective of the implementations of LM.

**Figure 11:**
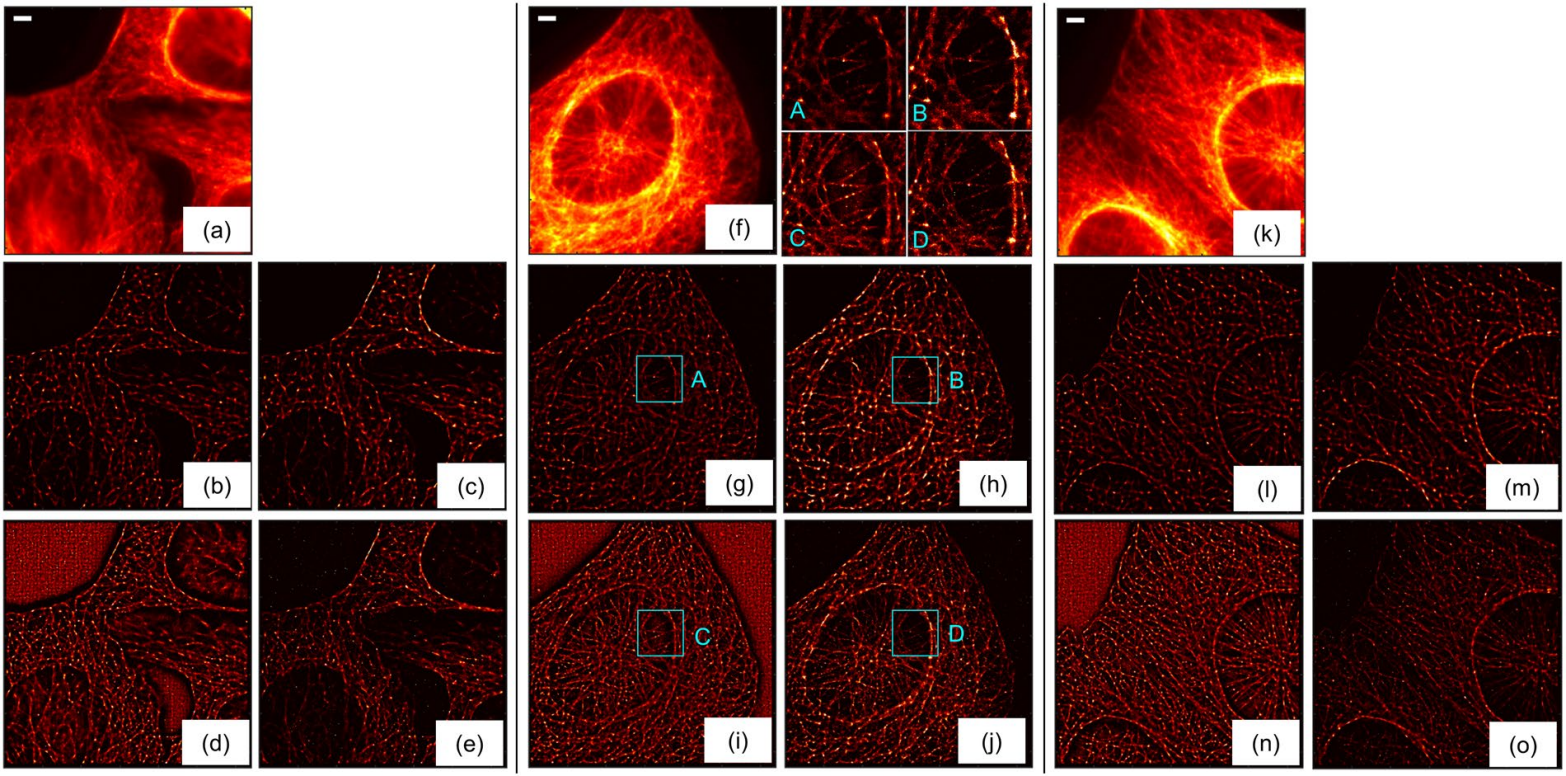
Results of original and debiased forms of LM in fixed cells are shown here. (**a**–**e**) Cell 1, (**f**–**j**) Cell 2, (k–o): Cell 3. (**a**,**f**,**k**) Mean image of 10000 frames, (**b**,**g**,**l**) Original LM using rainsSTORM, (**c**,**h**,**m**) debiased LM using rainsSTORM, (**d**,**i**,**n**) Original LM using NSTORM, and (**e**,**j**,**n**) Debiased LM using NSTORM. Scale bar: 2 um. The zoom-ins of squares A, B, C, D in (**g**,**h**,**i**,**j**) Are provided beside (**f**).

**Figure 12:**
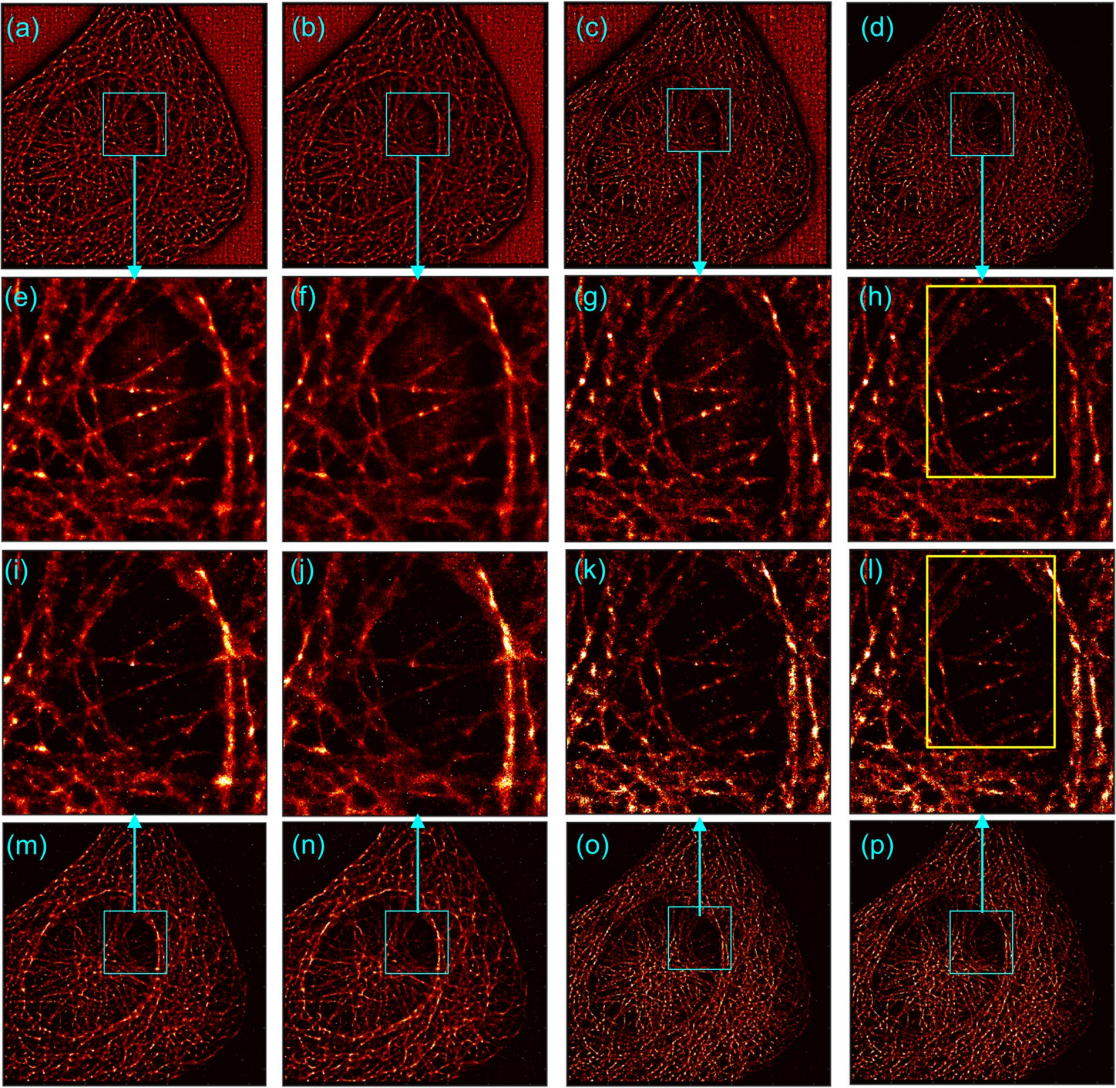
Debiasing as a non-heuristic and automatic way of constructing LM images while suppressing localizations of poor SNR is illustrated. (**a**,**e**,**i**,**m**) NSTORM results with no filters. (**b**,**f**,**j**,**n**) NSTORM results with localizations filtered on the filter 1, which is axial ratio of localization more than 1.3 as recommended in the manual of NSTORM. (**c**,**g**,**k**,**o**) NSTORM results with filter 1 and filter 2, which is the width of the Gaussian fit of localization being in the range (200, 400) nm, also recommended in NSTORM manual. (**d**,**h**,**l**,**p**) NSTORM results with filter 1, filter 2, and filter 3, which is the height of the Gaussian fit of localization being more than 100. (**a**–**d**) Original NSTORM image; (**e**–**h**) zoom-in of regions in cyan colored boxes in (**a**–**d**), respectively; (**m**–**p**) Debiased NSTORM images; (**i**–**l**) zoom-in of regions in cyan colored boxes in (**m**–**p**). Yellow boxes in (**h**,**l**) are used in Fig. 13.

We use rainSTORM and NSTORM implementations, rainSTORM being an open source implementation of least squares based localization and incapable of separating two molecules that are within diffraction limit and emitting simultaneously. The exact details of NSTORM are unavailable due to the proprietary nature of the implementation; the documentation of NSTORM indicates only that the localizations are obtained by Gaussian fit and that NSTORM allows localization of molecules with overlapping peaks. We used a thorough segmentation of regions of interest in both the implementations and the same value of radius of PSF. We note that the default setting of NSTORM does not use a thorough search and employs filters for selecting better localization. The default configuration of NSTORM thus provides better reconstruction and generates lesser artifacts such as granulated background observed in Fig. 11(d,i,n). The specific details regarding segmentation in NSTORM are unavailable. But, the filters of NSTORM in the general context of LM are discussed later through Fig. 12 and compared with debiased NSTORM results.

The mean images of the three datasets are shown in Fig. 11(a,f,k). The original reconstructions of rainSTORM and NSTORM are given in Fig. 11(b,g,l) and (d,i,n), respectively. NSTORM shows artifacts in the background due to the post-processing filters toggled off in executing NSTORM. Such artifacts or background may be introduced in the super-resolved image either due to computational aspects of specific implementations of LM as well as due to population of diffusing emitters or other sample dynamics. The debiased rainSTORM and debiased NSTORM reconstructions are shown in Fig. 11(c,h,m) and (e,j,o), respectively. Not only the artifacts in the original NSTORM results are suppressed due to debiasing, the debiased images of both implementations provide better mutual agreement in the structural details. The insets for the results of the second dataset further affirm the structural agreement after debiasing.

**Figure 13:**
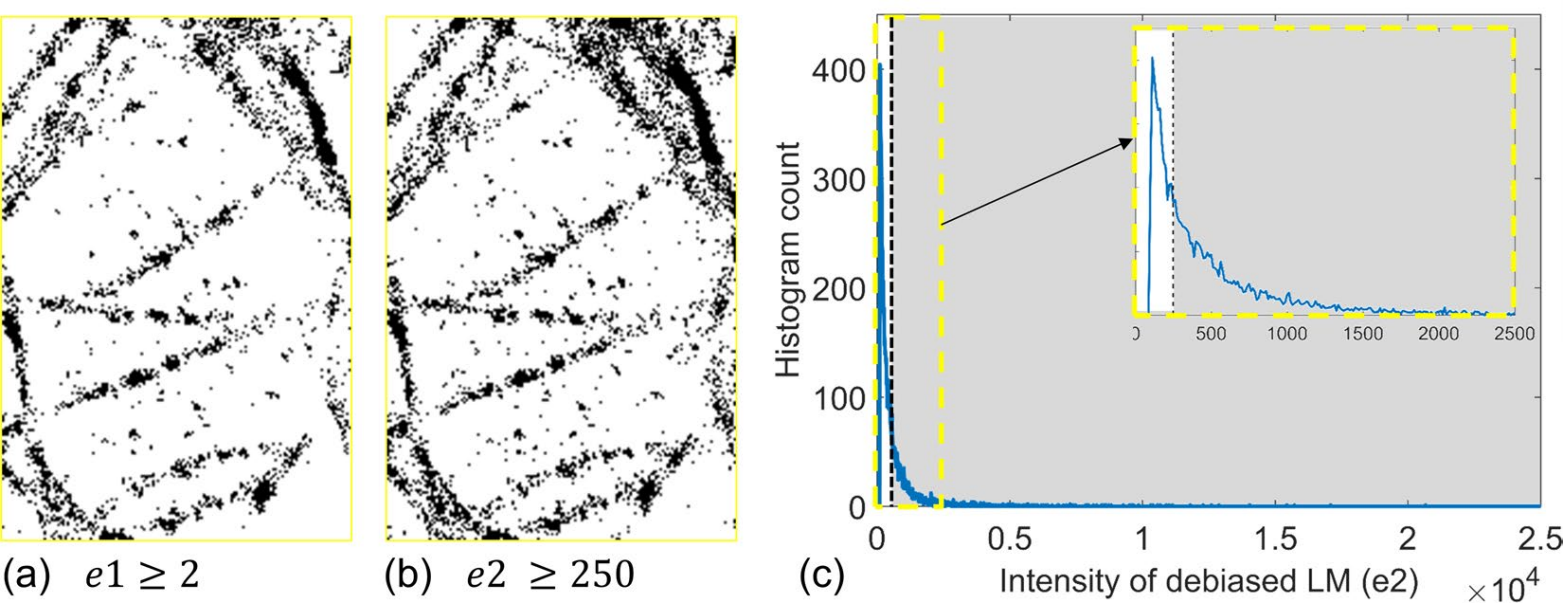
Binary segmentations of yellow boxes in Fig. 12(h,l) show that debiasing does not miss details. (**a**) Shows binary segmentation of the original LM using threshold on the original LM intensities (*e*1). (**b**) Shows binary segmentation of the debiased LM using threshold on the debiased LM intensities (*e*2), whose histogram and the heuristically chosen threshold are shown in (**c**). Details about histograms are in Supplementary Note 5.

As discussed before, heuristic filters are commonly used for selecting the localizations used for constructing LM images as an indirect way to suppress poor localizations due to poor SNR. Examples of such filters include thresholding on the axial ratio of the fitted 2-dimensional PSF (referred to as axial ratio), width of the fitted PSF, and the height of the Gaussian fit of localization. Often a combination of these is employed, including in NSTORM. We consider the example of cell 2 and show the results of NSTORM images with such filters in Fig. 12. It is seen that the debiased NSTORM image (Fig. 12(i,m)) without using any filter is qualitatively comparable with the most filtered NSTORM image (Fig. 12(d,h)). Debiasing of filtered NSTORM results (Fig. 12(j–l,n–p)) provides marginal improvements over the debiased NSTORM image of unfiltered localizations (Fig. 12(i,m)). Thus, the utility of proposed non-heuristic debiasing technique for LM as an automated way of giving preference to good localizations while not rejecting poor localizations completely is demonstrated. We also note through Fig. 13 that debiasing does not miss localizations. It simply modulates the weight of each localization and provides flexibility of segmentation, as demonstrated in Fig. 10.

## Conclusions

This work investigates the role of temporal variations in SNR in the state-of-the-art LM and MUSICAL techniques. It is demonstrated that the conventional form of LM over-represented the frames with lower SNR while MUSICAL under-represents the frames with lower SNR. To the best of our knowledge, such bias is identified explicitly and treated for the first time. We present techniques for debiasing LM and MUSICAL which show effectiveness in compensating for this bias without deteriorating the structural resolution of LM and MUSICAL. Importantly, we show that our debiasing technique for LM is a good candidate for introducing automated, non-heuristic, and soft weighing of localizations in comparison to the conventional retain-or-reject approach based on heuristically defined filters. We also present an insight into these techniques and identify the conditions in which they can be effective. We note that the debiasing techniques presented here are not they only techniques and may not be the best techniques for debiasing LM and MUSICAL. We recognize that more work is needed for finding elegant solutions that completely overcome such bias.

## Electronic supplementary material

Supplemenary Information

## References

[1] Rust MJ, Bates M, Zhuang X (2006). Sub-diffraction-limit imaging by stochastic optical reconstruction microscopy (STORM). Nature Methods.

[2] Betzig E (2006). Imaging intracellular fluorescent proteins at nanometer resolution. Science.

[3] Hess ST, Girirajan TP, Mason MD (2006). Ultra-high resolution imaging by fluorescence photoactivation localization microscopy. Biophysical Journal.

[4] Sharonov A, Hochstrasser RM (2006). Wide-field subdiffraction imaging by accumulated binding of diffusing probes. Proceedings of the National Academy of Sciences.

[5] Heilemann M (2002). High-resolution colocalization of single dye molecules by fluorescence lifetime imaging microscopy. Analytical Chemistry.

[6] Small A, Stahlheber S (2014). Fluorophore localization algorithms for super-resolution microscopy. Nature Methods.

[7] Agarwal, K. & Macháň, R. Multiple signal classification algorithm for super-resolution fluorescence microscopy. *Nature Communications* **7** (2016). 10.1038/ncomms13752 PMC5155148 27934858

[8] Agarwal, K. & Prasad, D. K. Eigen-analysis reveals components supporting super resolution imaging of blinking fluorophores. *Scientific Reports* **7** (2017). 10.1038/s41598-017-04544-5 PMC5493635 28667336

[9] Cox S (2012). Bayesian localization microscopy reveals nanoscale podosome dynamics. Nature Methods.

[10] Zhu L, Zhang W, Elnatan D, Huang B (2012). Faster STORM using compressed sensing. Nature Methods.

[11] Holden SJ, Uphoff S, Kapanidis AN (2011). DAOSTORM: an algorithm for high-density super-resolution microscopy. Nature Methods.

[12] Huang F, Schwartz SL, Byars JM, Lidke KA (2011). Simultaneous multiple-emitter fitting for single molecule super-resolution imaging. Biomedical Optics Express.

[13] Ashida Y, Ueda M (2016). Precise multi-emitter localization method for fast super-resolution imaging. Optics Letters.

[14] Gustafsson N (2016). Fast live-cell conventional fluorophore nanoscopy with ImageJ through super-resolution radial fluctuations. Nature Communications.

[15] Small A (2016). Multifluorophore localization as a percolation problem: limits to density and precision. Journal of the Optical Society of America A.

[16] Reiger B, Nieuwenhuizen R, Stallinga S (2015). Image processing and analysis for single molecule localization microscopy: Computation for nanoscale imaging. IEEE Signal Processing Magazine.

[17] Rees EJ, Erdelyi M, Schierle GSK, Knight A, Kaminski CF (2013). Elements of image processing in localization microscopy. Journal of Optics.

[18] Biomedical Imaging Group. Ecole Polytechnique Fédérale de Lausanne. Switzerland. Single Molecule Localization Microscopy Symposium Challenge 2016. http://bigwww.epfl.ch/smlm/challenge2016/ (2016).

[19] Rees EJ (2012). Blind assessment of localisation microscope image resolution. Optical Nanoscopy.

[20] Dempsey GT, Vaughan JC, Chen KH, Bates M, Zhuang X (2011). Evaluation of fluorophores for optimal performance in localization-based super-resolution imaging. Nature Methods.

[21] Thompson RE, Larson DR, Webb WW (2002). Precise nanometer localization analysis for individual fluorescent probes. Biophysical Journal.

[22] Ober RJ, Ram S, Ward ES (2004). Localization accuracy in single-molecule microscopy. Biophysical Journal.

[23] Smith CS, Joseph N, Rieger B, Lidke KA (2010). Fast, single-molecule localization that achieves theoretically minimum uncertainty. Nature Methods.

[24] Nahidiazar, L., Agronskaia, A. V., Broertjes, J., van den Broek B. & Jalink, K. Optimizing imaging conditions for demanding multi-color super resolution localization microscopy. *PloS One* **11** (2016). 10.1371/journal.pone.0158884 PMC4938622 27391487

[25] Reuss, M. *et al*. Measuring true localization accuracy in super resolution microscopy with DNA-origami nanostructures. *New Journal of Physics* **19** (2017).

[26] Andronov. L., Orlov, I., Lutz, Y., Vonesch, J. L. & Klaholz, B. P. ClusterViSu, a method for clustering of protein complexes by Voronoi tessellation in super-resolution microscopy. *Scientific reports* **6** (2016). 10.1038/srep24084 PMC4828638 27068792

[27] Otsu N (1975). A threshold selection method from gray-level histograms. Automatica.

[28] Sezgin M, Sankur B (2004). Survey over image thresholding techniques and quantitative performance evaluation. Journal of Electronic Imaging.

[29] Wang Z, Bovik AC, Sheikh HR, Simoncelli EP (2004). Image quality assessment from error visibility to structural similarity. IEEE Transactions on Image Processing.

