# Supplementary material for: Non-heuristic automatic techniques for overcoming low signal-to-noise-ratio bias of localization microscopy and multiple signal classification algorithm
Source: Sci Rep. 2018 Mar 21;8:4988. doi: 10.1038/s41598-018-23374-7 (PMC5862973; doi:10.1038/s41598-018-23374-7)
Supplement: Supplementary file 1 — Supplemenary Information [file 41598_2018_23374_MOESM1_ESM.pdf]

Non-heuristic automatic techniques for overcoming low signal-to-noise-ratio bias of localization microscopy and multiple signal classification algorithm

- Supplementary information

Krishna Agarwal[1], Radek Macháň[2,3], and Dilip K. Prasad[4]

[1]Department of Physics and Technology, UiT-The Arctic University of Norway, 9037 Tromsø, Norway

[1]uthkrishth@gmail.com

[2]National University of Singapore, Singapore

[3]Faculty of Science, Charles University in Prague, Czech Republic

[2]radek82machan@gmail.com

[4]School of Computer Science and Engineering, Nanyang Technological University, Singapore

[4]dilipprasad@gmail.com

Supplementary Note 1. Temporal patterns in fluorescence intensity in response to natural or experimentally introduced changes in photokinetics of fluorophores

It is commonly assumed that the fluorescence characteristics of the emitters do not change within the timescale of the acquisition of images for localization microscopy. The overall decrease in intensity is often attributed to the decrease in emitter density due to irreversible loss of some of them through bleaching. This is easily verified in Supplementary Figure 1 through the synthetic example of fork in which bleaching is simulated as described in methods. Other characteristics of the emitters remain constant. The decrease in the number of fluorescing emitters shown in Supplementary Figure 1(d) translates to the decrease in image intensity shown in Supplementary Figure 1(b), whereas the average of the actual number of photon emissions remains relatively flat through the image acquisition, as shown in Supplementary Figure 1(c). In Supplementary Figure 1(c), we show the histogram of the number of photons emitted by the fluorophores in each batch of 100 frames. The vertical axis shows the histogram bins, the horizontal axis corresponds to the frame number and the color bar indicates the number of localized fluorophores in a histogram bin. Points on the cyan line show the mean of the photon emissions in individual batches.

We illustrate other examples of changes in intensity over time due to chemical changes and external intervention using datasets of microtubules in cells. Here, due to the absence of ground truth, the number of localizations or photons correspond to the estimated numbers. Further, the quantity plotted as estimated number of photons is actually linearly proportional to the estimated number of photons, the unknown constant of proportionality being the characteristic of the measurement system and computation algorithm. We use the estimations of both rainSTORM and NSTORM for a more conclusive inference.

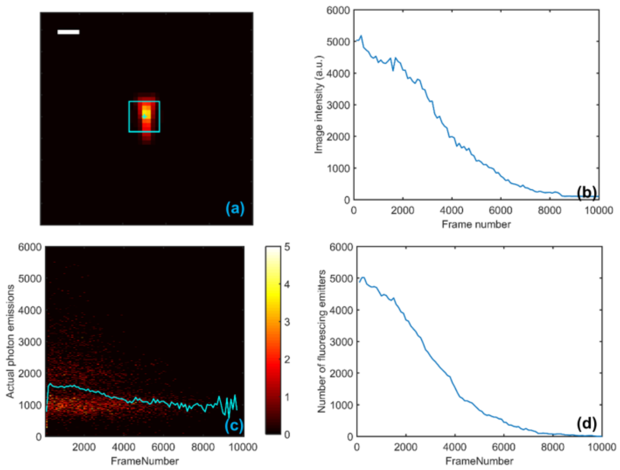

Supplementary Figure 1. Illustration of decrease in intensity over time due to photobleaching of emitters. The statistics are calculated in batches of 100 frames each. Scale bar in (a) is 500 nm. The intensity plot in (b) is for the cyan point at the center of the square shown in (a). The statistics in (c-d) are computed for the pixels in the square region identified in (a). Points on the cyan line in (c) show the mean of the photon emissions in individual batches.

Beside photobleaching, another reason of patterned decrease in fluorescence intensity may be the variations in the fluorescence emission efficiencies (mostly through differences in the fraction of time they spend in the on-state) potentially due to change in concentration of chemicals influencing photokinetic phenomena [S2]. This appears to be the case with the dataset of microtubules in cell 2. The

image intensity shown in Supplementary Figure 2(b) indicates decline with time. However, the number of localizations does not show a declining trend, neither in NSTORM (Supplementary Figure 2(d)) nor in rainSTORM (Supplementary Figure 2(f)), thus indicating the potential absence of photobleaching. On the other hand, the number of photon emissions shows a clear trend of decline in both NSTORM (Supplementary Figure 2(c)) and rainSTORM (Supplementary Figure 2(e)).

Sometimes, in order to improve blinking or fluorescence intensity, manual intervention may be done mid-way in the form of adding certain chemicals, increasing the excitation power, or switching on an additional activation source. In datasets of microtubules in cells 1 and 3, an activation laser was switched on mid-way when the incidents of blinking or their intensity was heuristically concluded as quite diminished. Upon further incident of diminished blinking or intensity, the power of the activation laser was increased. This results in sudden increase in the fluorescence intensity, as observed in Supplementary Figure 3(b) and Supplementary Figure 4(b). It is also clearly observed in the histogram of photon emissions in Supplementary Figure 3(c,e) and Supplementary Figure 4(c,e). Thus, evidently, there is a temporal variation in the fluorescence intensity, which translates into variation in the signal to noise ratio owing to the shot noise characteristics of cameras.

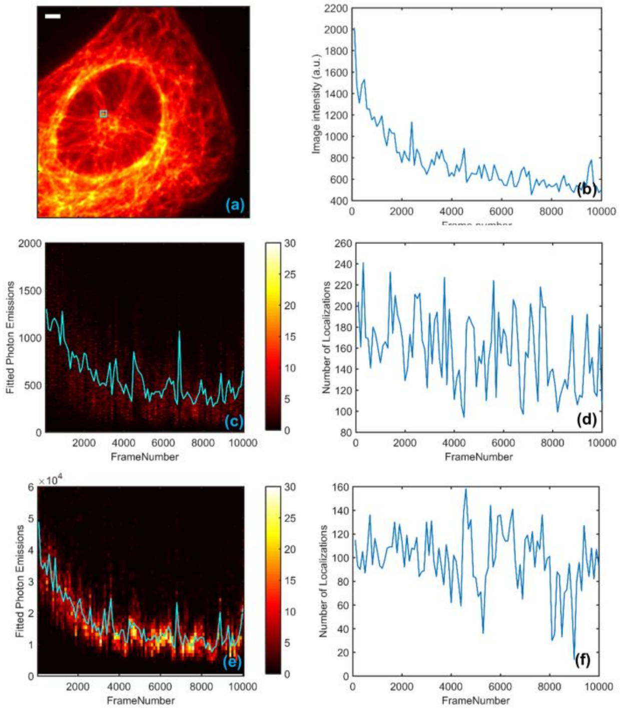

Supplementary Figure 2. Illustration of change in intensity over time potentially due to chemical changes in the buffer. The statistics are calculated in batches of 100 frames each. Scale bar in (a) is 2 um. The intensity plot in (b) is for the cyan point at the center of the square shown in (a). The statistics in (c-f) are computed for the pixels in the square region identified in (a). Points on the cyan line in (c) show the mean of the photon emissions in individual batches. (c,d): NSTORM; (e,f): rainSTORM.

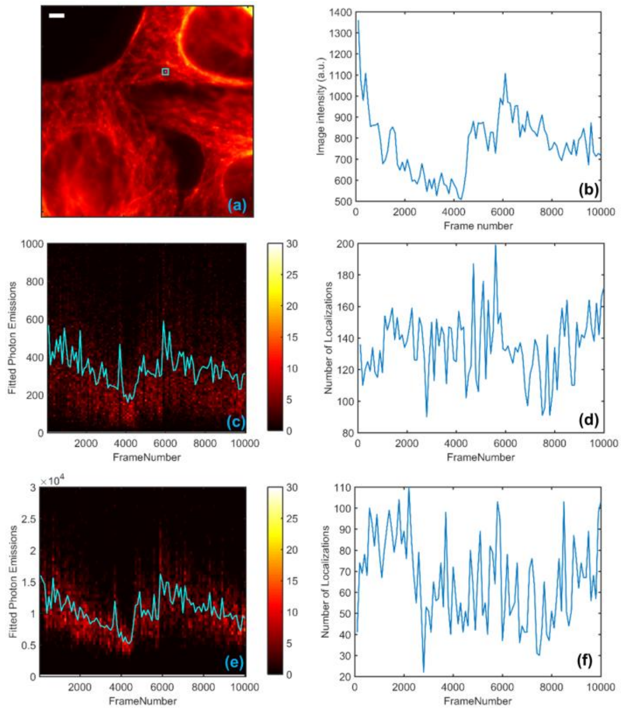

Supplementary Figure 3. Illustration of change in intensity over time due to switching on of an activation laser and increasing its power. The statistics are calculated in batches of 100 frames each. Scale bar in (a) is 2 um. The intensity plot in (b) is for the cyan point at the center of the square shown in (a). The statistics in (c-f) are computed for the pixels in the square region identified in (a). Points on the cyan line in (c) show the mean of the photon emissions in individual batches. (c,d): NSTORM; (e,f): rainSTORM.

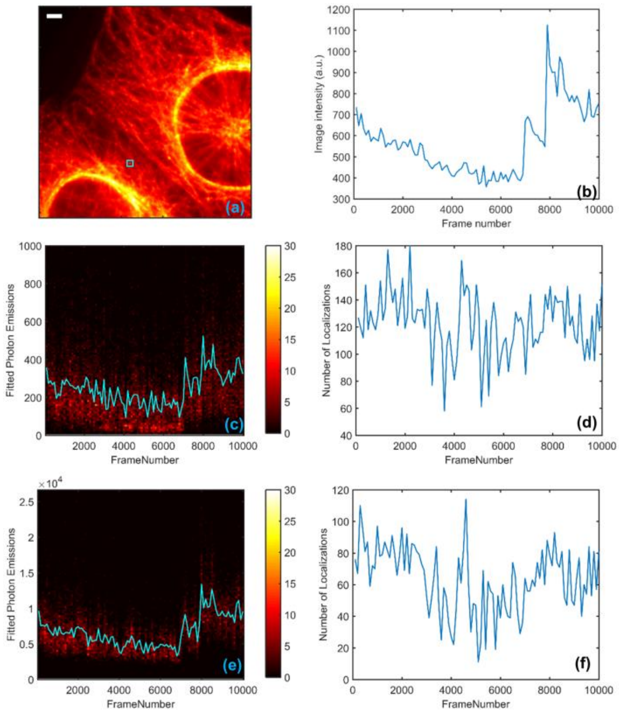

Supplementary Figure 4. Illustration of change in intensity over time due to switching on of an activation laser and increasing its power. The statistics are calculated in batches of 100 frames each. Scale bar in (a) is 2 um. The intensity plot in (b) is for the cyan point at the center of the square shown in (a). The statistics in (c-f) are computed for the pixels in the square region identified in (a). Points on the cyan line in (c) show the mean of the photon emissions in individual batches. (c,d): NSTORM; (e,f): rainSTORM.

Supplementary Note 2. Detection of foreground in a frame

Consider an image frame $\mathbf{I}_k$ which contains intensities at all the pixels in the kth frame (see Supplementary Figure 5 (a)). The histogram of its image intensity (HoI) is computed as $h_k(n)$, where n is a discrete intensity value and $h_k(n)$ is the number of pixels with intensity value n in the image $\mathbf{I}_k$. In images of sparsely emitting emitters, the foreground pixels are so few that the histogram distribution essentially appears unimodal and corresponds to background (see Supplementary Figure 5 (b)). In fact, the number of foreground pixels is typically orders of magnitudes smaller than the number of background pixels. Thus, we consider logarithm of HoI (LoHoI), $g_k(n) = \log(k_k(n))$. We empirically found that even $g_k(n)$ is not amenable for identifying and separating the bimodal distributions (see Supplementary Figure 5 (c)). However, the values of LoHoI have a bimodal distribution. The threshold value which separates these two distributions is computed using the popular Otsu's method of thresholding [S1]. The thresholded LoHoI is used to find n_0 after which the thresholded LoHoI remains 0 (see Supplementary Figure 5 (c)). This value of n_0 is then used to obtain the foreground of the image (see Supplementary Figure 5 (d)).

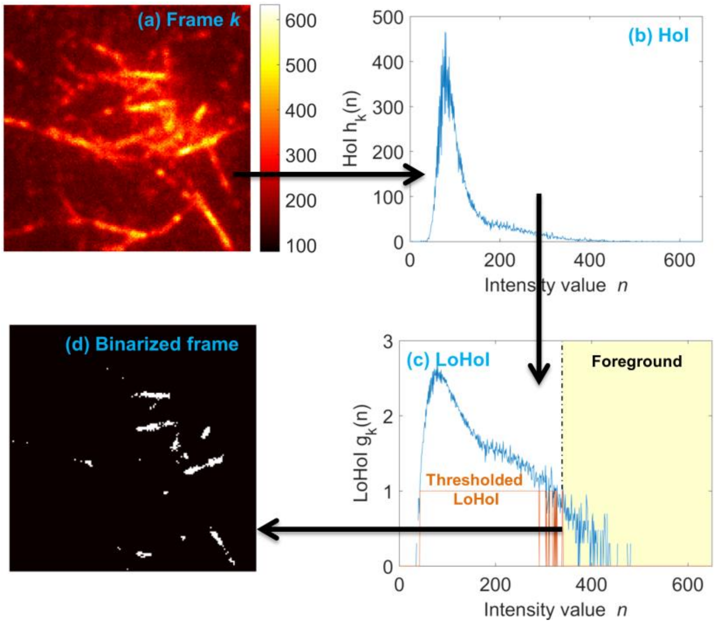

Supplementary Figure 5. Proposed method of foreground detection in an image frame. See Supplementary Note 2 for details

Supplementary Note 3. Other techniques for debiasing LM

Technique 2 for debiasing LM: Weighing contributions from the frame with its average intensity.

In this technique, $b_k(x, y)$ is defined as:

$$b_k(x, y) = \frac{\sum_{\forall r'} I(r')}{N_{r'}} \quad \text{(S.1)}$$

where r' denotes an image pixel in the measured image stack and $N_{r'}$ is the total number of pixels. In this technique, we penalize all the localizations in a frame equally, irrespective of the local spatial variations in SNR.

Technique 3 for debiasing LM: Weighing contributions from the frame with its average foreground intensity.

In this technique, $b_k(x, y)$ is defined as:

$$b_k(x, y) = \frac{\sum_{\forall r' \in F} I(x', y')}{N_{r'}} \quad \text{(S.2)}$$

where F is the foreground. Similar to technique 2, all localizations in a frame are penalized equally.

Technique 4 for debiasing LM: Weighing with inverse of localization accuracy.

In this technique, $b_k(x, y)$ is defined as:

$$b_k(x, y) = \frac{1}{\Delta_k(x, y)} \quad \text{(S.2)}$$

where $\Delta_k(x, y)$ is the localization accuracy of the localization at (x, y) in the k th frame. Here, we use the Thomson formula for computing $\Delta_k(x, y)$ [S3]. Similar to technique 1, each localization in a frame is treated individually and incorporates the effect of the local signal to background ratio on the quality of localization.

Comparison of the debiasing techniques of LM: The comparison of debiasing techniques 1-3 of LM is given in Supplementary Table 1 and Supplementary Figure 6. It is seen that the technique 1 is very effective for both the examples. We note that the techniques 1 and 4 give almost the same result. The SSIM values between the results of techniques 1 and 4 are equal to 99.4% for both the examples. The correlation between them is also very high, 97.4% for in-vitro actin filaments and 99.4% for in-vitro microtubules. Thus, it is evident that the number of estimated photons is highly correlated to inverse of localization precision. This is because the localization precision and estimated number of photons are both related to the signal to background ratio.

		Localization microscopy (original)	MUSICAL (original)	
In-vitro actin filaments				
Original	MUSICAL	88.48%	88.48%	Localization microscopy
Technique 1		90.60%	90.49%	
Technique 2		90.56%	88.02%	
Technique 3		90.76%	88.10%	
Technique 4		-	90.54%	
In-vitro microtubules				
Original	MUSICAL	87.15%	87.15%	Localization microscopy
Technique 1		87.19%	90.43%	
Technique 2		87.27%	87.81%	
Technique 3		85.60%	88.22%	
Technique 4		-	90.80 %	

Supplementary Table 1. Comparison of techniques of debiasing MUSICAL and LM.

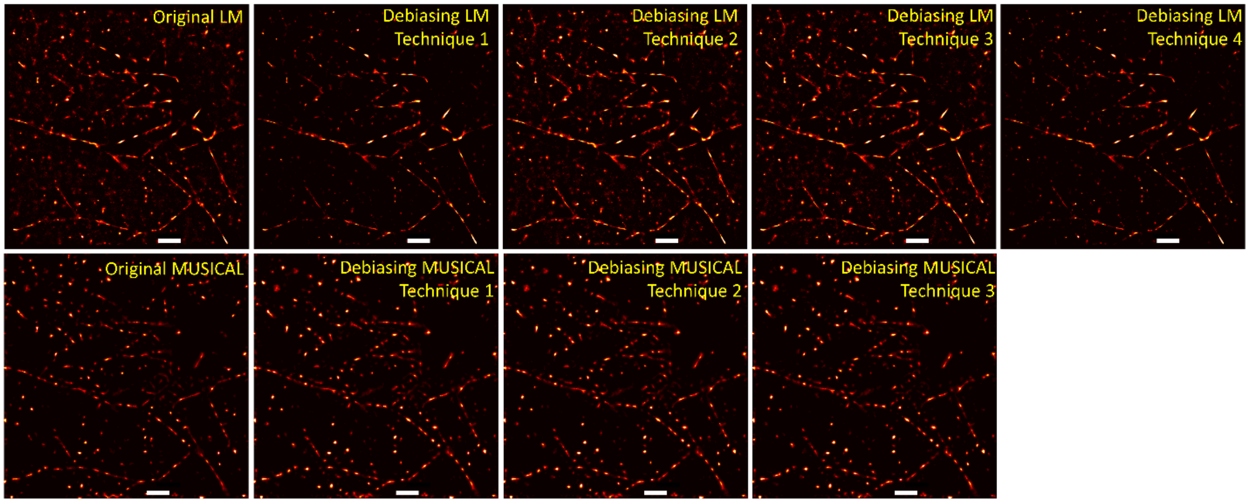

Supplementary Figure 6. Results of original and debiased MUSICAL and LM results for the example data of in-vitro actin filaments are provided here. Scale bars: 1 um.

Supplementary Note 4. Other techniques for debiasing MUSICAL

Technique 2 for debiasing MUSICAL: Image stack weighed by frames' average intensities. In this technique, $b_k(x, y)$ is defined as:

$$w_k = \left(\frac{\sum_{\forall r'} I(r')}{N_{r'}} \right)^{-1} \tag{S.3}$$

where r' denotes an image pixel in the measured image stack and $N_{r'}$ is the total number of pixels.

Technique 3 for debiasing MUSICAL: Image stack weighed by frames' standard deviation of intensities. Consider two images, one with high SNR and another with low SNR. The high SNR image exhibits high standard deviation spatially and vice versa. Thus, in this technique, $b_k(x, y)$ is defined as:

$$w_k = \left(\frac{\sum_{\forall r'} \left(I(r') - \tilde{I}_k \right)^2}{N_{r'}} \right)^{-1/2} \tag{S.4}$$

where r' denotes an image pixel in the measured image stack, $N_{r'}$ is the total number of pixels and $\tilde{I}_k$ is given as:

$$I_k = \frac{\sum_{\forall r'} I(r')}{N_{r'}} \tag{S.5}$$

Comparison of the debiasing techniques of MUSICAL: The comparison of debiasing techniques 1-3 of MUSICAL is given in Supplementary Table 1 and Supplementary Figure 6. It is seen that techniques 1 and 2 are comparable to each other. Technique 3 provides better value of SSIM for in-vitro actin filaments data but poorer value of SSIM for in-vitro microtubules. Thus, an inference regarding it cannot be made.

Supplementary Note 4. Histograms in Fig. 10 and 13 of the main paper.

In Fig. 10 (a), histogram of intensities of original LM image in Fig. 9(a) is given. The intensity of original LM image at a pixel is denoted as $s(x, y)$. Since the original image counts the number of localizations in a pixel in the LM image, the values of $s(x, y)$ are integers. Each of these integers is significantly less than the number of the frames because of the spatio-temporal sparsity of emissions. Fig. 10(a) shows the number of pixels in the original LM image at which an integer intensity value $e1$ is observed. Since the number of background pixels are too many and they contribute to the first bin only, we skip the first bin.

Fig. 10(b) and Fig. 13(c) show histogram of intensities of debiased LM image. The intensity of original LM image at a pixel is denoted as $s(x, y)$. Since the debiased image integrates the estimated number of photons emitted by localized emitter lying within a pixel in the LM image, the values of $s(x, y)$ are integers spanning very large range. The entire range of values taken by $s(x, y)$ be denoted as $e2 \in [0, a]$. Then, we create 10000 equal bins of span $a/10000$ in this range such that the outer edge of each bin is $\frac{(n-1)a}{10000}$, where n is the bin number. We count the number of pixels in debiased LM image whose intensity lies in these bins. We plot this count as the function of the outer edge of the bin. Since the number of background pixels are too many and they contribute to the first bin only, we skip the first bin.

References and links

[S1] N. Otsu, “A threshold selection method from gray-level histograms,” Automatica 11, 23–27 (1975).

[S2] L. Nahidiazar, A. V. Agronskaia, J. Broertjes, B. van den Broek, and K. Jalink, “Optimizing imaging conditions for demanding multi-color super resolution localization microscopy,” PloS One 11 (2016).

[S3] R. E. Thompson, D. R. Larson, and W. W. Webb, “Precise nanometer localization analysis for individual fluorescent probes,” Biophysical Journal 82, 2775–2783 (2002).
